# Immunogenicity and Safety of Monovalent Acellular Pertussis Vaccine at Birth: A Randomized Clinical Trial

**DOI:** 10.1001/jamapediatrics.2018.2349

**Published:** 2018-09-10

**Authors:** Nicholas Wood, Terry Nolan, Helen Marshall, Peter Richmond, Emma Gibbs, Kirsten Perrett, Peter McIntyre

**Affiliations:** 1 National Centre for Immunisation Research and Surveillance of Vaccine Preventable Diseases, Westmead, New South Wales, Australia; 2 The Children’s Hospital at Westmead, Sydney, New South Wales, Australia; 3 The University of Sydney, Sydney, New South Wales, Australia; 4 School of Population and Global Health, University of Melbourne, Melbourne, Victoria, Australia; 5 Murdoch Children’s Research Institute, Melbourne, Victoria, Australia; 6 Vaccinology and Immunology Research Trials Unit, Women’s and Children’s Health Network, North Adelaide, South Australia, Australia; 7 Robinson Research Institute and Adelaide Medical School, The University of Adelaide, Adelaide, South Australia, Australia; 8 University of Western Australia, Division of Paediatrics and Vaccine Trials Group, Wesfarmers Centre of Vaccines and Infectious Diseases, Telethon Kids Institute, Perth, Western Australia, Australia; 9 National Health and Medical Research Council Clinical Trials Centre, University of Sydney, Camperdown and Darlington, New South Wales, Australia; 10 The Royal Children’s Hospital, Melbourne, Victoria, Australia

## Abstract

**Importance:**

An alternative option to maternal vaccination to prevent severe pertussis in infants is vaccination at birth. Data are needed on the immunogenicity and safety of a birth dose of monovalent acellular pertussis (aP) vaccine.

**Objective:**

To compare IgG antibody responses to vaccine antigens at 6, 10, 24, and 32 weeks of age between newborn infants receiving the aP vaccine and hepatitis B vaccine (HBV) or HBV alone.

**Design, Setting, and Participants:**

A randomized clinical trial was conducted at 4 sites in Australia (Sydney, Melbourne, Adelaide, and Perth) between June 11, 2010, and March 14, 2013, among 440 healthy term (>36 weeks’ gestation) infants aged less than 5 days at recruitment. Statistical analysis was performed from March 1, 2015, to June 2, 2016.

**Intervention:**

Newborns received HBV and, after stratification by maternal receipt of adult-formulated aP-containing vaccine (tetanus toxoid, reduced diphtheria toxoid, and pertussis antigen content [Tdap]) prior to pregnancy, were block randomized to receive the aP vaccine (without diphtheria or tetanus) within 5 days of birth or not. At 6, 16, and 24 weeks, infants received a hexavalent vaccine with pediatric-formulated diphtheria, tetanus and pertussis antigens (DTaP), *Haemophilus influenzae* type b (Hib), HBV, and polio vaccine, as well as the 10-valent pneumococcal conjugate vaccine.

**Main Outcomes and Measures:**

Detectable (>5 enzyme-linked immunosorbent assay units per milliliter) and geometric mean concentrations of IgG antibody to pertussis toxin (PT), pertactin, and filamentous hemagglutinin at 6, 10, and 24 weeks stratified by maternal Tdap history, and antibody at 32 weeks to HBV, Hib, polio, diphtheria, tetanus, and pneumococcal serotypes. The primary outcome was detectable IgG to both PT and pertactin at 10 weeks.

**Results:**

A total of 440 infants (207 girls and 233 boys; median gestation, 39.2 weeks) were randomized to receive the aP vaccine plus HBV (n = 221) or HBV only (control group; n = 219). At 10 weeks, 192 of 206 infants who received the aP vaccine (93.2%) had detectable antibodies to both PT and pertactin vs 98 of 193 infants in the control group (50.8%) (*P* < .001), with the geometric mean concentration for PT IgG 4-fold higher among the group that received the aP vaccine. At age 32 weeks, all infants (n = 181 with sera available for testing) who received the aP vaccine at birth had detectable PT IgG and significantly lower IgG geometric mean concentrations for Hib, hepatitis B, diphtheria, and tetanus antibodies. Local and systemic adverse events were similar between both groups at all time points.

**Conclusions and Relevance:**

The monovalent aP vaccine is immunogenic and safe in neonates and, if licensed and available, would be valuable for newborns whose mothers did not receive the Tdap vaccine during pregnancy.

**Trial Registration:**

http://anzctr.org.au Identifier: ACTRN12609000905268

## Key Points

**Question:**

Is the acellular pertussis vaccine immunogenic and safe when given at birth?

**Findings:**

In this randomized clinical trial, administration of the acellular pertussis vaccine at birth resulted in higher pertussis antibody responses at 6 and 10 weeks of age, with postprimary responses equivalent to those in the control group and no difference in systemic or local reactions. Administration of the acellular pertussis vaccine at birth resulted in nonsignificant reductions in antibody responses to some concomitantly administered antigens.

**Meaning:**

Administration of the acellular pertussis vaccine at birth has the potential to reduce severe morbidity from *Bordetella pertussis* infection in the first 3 months of life, especially for infants of mothers who have not received a pertussis vaccine during pregnancy.

## Teaser

This randomized clinical trial compares IgG antibody responses to vaccine antigens at age 6, 10, 24, and 32 weeks between newborn infants receiving the monovalent acellular pertussis vaccine and the hepatitis B vaccine and those receiving the hepatitis B vaccine alone.

## Introduction

In developed countries, deaths from pertussis in the prevaccine era occurred during the first 2 years of life, but deaths from pertussis in the postvaccine era have been largely restricted to unvaccinated infants younger than 8 weeks of age.^1,2^ One infant dose of pertussis vaccine provides significant protection against death.^3,4,5^ The high incidence of death from pertussis in the first 3 months of life prompted early studies of vaccination of the mother during pregnancy and of the infant at birth.^6,7^ Although the first neonatal trials, using whole-cell pertussis vaccines, were undertaken in the 1940s, later concerns about immune tolerance^6,7^ and reduced responses in the presence of maternal antibody^8^ discouraged further study until acellular vaccines became available. A study of the administration of pediatric-formulated tetanus toxoid, diphtheria toxoid, and acellular pertussis vaccine (DTaP) at birth suggested impaired pertussis antibody responses at 6 months,^9^ but studies of the monovalent aP vaccine without diphtheria and tetanus^10,11,12^ found favorable responses.

At the inception of this study in 2009, when the administration of tetanus toxoid, reduced diphtheria toxoid, and pertussis antigen content (Tdap) in pregnancy was deemed problematic owing to legal and attitudinal barriers,^13,14^ we set out to test the potential for the administration of the aP vaccine at birth being implemented more widely. In 2009, as postpartum administration of Tdap had been routinely recommended in Australia and the United States for some years, it was important that responses to neonatal aP vaccination be assessed with reference to prepartum Tdap. Therefore, we designed a study to detect clinically meaningful immunogenicity and safety end points, including purposeful recruitment of a subset of mothers who had documented receipt of a Tdap within 5 years prior to delivery, to assess infant responses following aP vaccine at birth.^15,16^

## Methods

### Study Design and Participants

This phase 3 randomized, nonblinded clinical trial of the administration of monovalent aP vaccine to newborns was conducted between June 11, 2010, and March 14, 2013, in 4 cities in Australia (Sydney, Melbourne, Perth, and Adelaide). The trial protocol is available in Supplement 1. Appropriate regulatory and ethical approval was granted by the Clinical Trial Notification Scheme, Therapeutic Goods Administration, Department of Health and Ageing, Australian Government; Sydney Children’s Hospitals Network Human Research Ethics Committee; Royal Children’s Hospital, Melbourne Human Research Ethics Committee; Women’s and Children’s Hospitals, Adelaide, Human Research Ethics Committee; and Princess Margaret Hospital for Children Human Research Ethics Committee. The trial was registered on the Australian New Zealand Clinical Trials Registry and was reported using CONSORT guidelines. Written informed consent was obtained from parents or guardians before the enrollment of infants.

Mothers of eligible infants were approached in antenatal clinics or postnatal wards in participating hospitals. Eligible participants were healthy infants born at least at 36 weeks’ gestation after an uncomplicated pregnancy to mothers who were seronegative for hepatitis B surface antigen. Infants were enrolled within 5 days (120 hours) of birth. Enrollment in the study was excluded by known contraindications to vaccination,^17^ including any confirmed or suspected immunosuppressive or immunodeficiency condition in the parent or child and any major congenital defects or serious chronic illness.

### Randomization and Blinding

Mothers of eligible infants were stratified into those who reported receipt of Tdap or had a laboratory-confirmed pertussis infection within 5 years of delivery (but not during pregnancy) or infants born to women who did not report receipt of Tdap or had a laboratory-confirmed pertussis infection within 5 years of delivery. Confirmation of self-reported maternal Tdap vaccination status was later sought from the primary health care professional who administered the vaccine. After stratification, infants were randomized in a 1:1 ratio using an internet-based randomization system (Interactive Voice Response System; National Health and Medical Research Council Clinical Trials Centre, Sydney). This was an open-label study; study personnel and parents knew which vaccines were being administered.

#### Intervention

At visit 1, neonates received the aP vaccine and the hepatitis B vaccine (HBV) in opposite thighs within 120 hours of birth (the aP group) or received only HBV (the control group). At age 6, 16, and 24 weeks, all infants in all sites then received a hexavalent vaccine routinely administered at that time in Australia (DTaP–hepatitis B–*Haemophilus influenzae* type b–inactivated poliovirus) and the 10-valent pneumococcal conjugate vaccine. Infants at the Sydney site received the oral rotavirus vaccine at age 6 and 16 weeks and those at the other sites received the oral rotavirus vaccine at age 6, 16, and 24 weeks. Details on the vaccines used are provided in the eAppendix in Supplement 2.

#### Immunology

The first serum sample was obtained from the mother at enrollment of her infant (visit 1; within 120 hours of birth). No sample of umbilical cord blood was collected. Subsequent sera samples (n = 4) were collected from infants at 6 weeks (visit 2), 10 weeks (visit 3), 24 weeks (visit 5), and 32 weeks of age (visit 6) (Figure). Details on laboratory analysis and serology measurement are provided in the eAppendix in Supplement 2.

**Figure.**
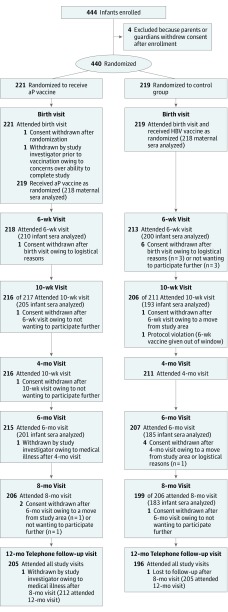
Flowchart of Study Population The numbers of infants approached, assessed for eligibility, and primarily excluded are unknown. aP indicates acellular pertussis; HPV, hepatitis B vaccine.

#### Safety

After administration of each vaccine, all infants were observed for 30 minutes. Vaccine reactogenicity and safety were assessed using a 7-day diary card after each vaccination. Details on adverse event data collection are provided in the eAppendix in Supplement 2.

### Main Outcome Measures

#### Primary End Point

The primary end point was the proportion of infants with IgG antibody responses to both pertussis toxin (PT) and pertactin (PRN) greater than 5 enzyme-linked immunosorbent assay units per milliliter (ELU/mL) at 10 weeks. This primary outcome was chosen based on human household contact studies.^18,19^

#### Secondary End Points

Infants’ IgG antibody responses to PT, PRN, and filamentous hemagglutinin (FHA) collected at 6, 24, and 32 weeks of age were compared according to receipt of aP vaccine at birth or not, stratified by confirmed maternal Tdap vaccination or pertussis infection and the presence of detectable maternal pertussis antibodies at birth. The IgG antibody responses to other antigens (hepatitis B, Hib, polio, diphtheria, tetanus, and pneumococcal) measured at 32 weeks were compared as already described.

### Statistical Analysis

Statistical analysis was performed from March 1, 2015, to June 2, 2016. All serum antibody concentrations were log transformed for statistical analysis as geometric mean concentrations (GMCs). Where protective thresholds are well established, the proportions at or above this threshold (antidiphtheria, >0.1 IU/mL; antitetanus, >0.1 IU/mL; anti-Hib, >0.15 μg/mL; and hepatitis B surface antibody, >10 and >100 mIU/mL) were compared by vaccine group. Statistical analysis includes both comparisons of GMC (with 95% CIs) as a continuous variable (*t* test) and categorical analysis of relevant antibody thresholds (χ^2^ test). For the primary outcome, comparisons between treatment groups were performed using a Cochran-Mantel-Haenszel χ^2^ test stratified by maternal Tdap status and maternal pertussis infection status, yielding an estimate of the odds ratio and associated 95% CI. The treatment effect was expressed as differences in proportion. Statistical analysis was performed at the National Health and Medical Research Council Clinical Trials Centre using SAS, version 9.3 (SAS Institute Inc), independent of the testing laboratory. All *P* values were from 2-sided tests, and results were deemed statistically significant at *P* < .05.

## Results

A total of 444 participants were registered, of whom 440 were randomized and 417 (94.8% [212 in the aP group and 205 in the control group]) completed the study (Figure). A total of 96 infants (49 in the aP group and 47 in the control group) were born to mothers with documented receipt of Tdap within 5 years prior to delivery, with 1 mother in each group with laboratory-confirmed pertussis infection within 5 years prior to delivery. The median age of the mothers was 33.6 years in the aP group and 33.4 years in the control group. The median gestation of the infants was 39.2 weeks in both groups; 207 infants were girls, and 233 were boys; and 370 infants (84.1%) were white (189 of 221 enrolled in the aP group [85.5%] and 181 of 219 enrolled in the control group [82.6%]). Receipt of the aP vaccine was within 2 days of birth for 96 of 221 patients enrolled (43.4%) and between 3 and 5 days of birth for 124 of 221 patients enrolled (56.1%); 55 of 221 patients enrolled (24.9%) received HBV and the aP vaccine on the same day (Table 1). No infants received the Bacillus Calmette-Guérin vaccine at birth.

**Table 1.** Characteristics of Study Participants According to Group

Characteristic	aP Group (n = 221)	Control Group (n = 219)
Birth weight, mean (range), g	3479.0 (3417.6-3540.5)	3548.8 (3492.7-3605.0)
Gestation, mean (range), wk	39.2 (39.0-39.4)	39.2 (39.0-39.3)
Boys, No. (%)	117 (52.9)	116 (53.0)
aP vaccine days 0-2, No. (%)	96 (43.4)	NA
aP vaccine days 3-5, No. (%)	124 (56.1)	NA
HBV days 0-2, No. (%)	201 (91.0)	191/218 (87.6)
HBV days 3-5, No. (%)	20 (9.0)	27/218 (12.4)
Withdrew prior to 2 mo of age, No. (%)	3 (1.4)	5 (2.3)
Documented maternal Tdap within 5 y prior to enrollment, No. (%)	49 (22.2)	47 (21.5)

Abbreviations: aP, monovalent acellular pertussis vaccine; HBV, hepatitis B vaccine; NA, not applicable; Tdap, tetanus toxoid, reduced diphtheria toxoid, and pertussis antigen content.

### Immunogenicity

#### Antibody Responses to Pertussis Vaccination

##### Primary Outcome

Infants who received the aP vaccine within 5 days of birth were significantly more likely to have IgG antibody to both PT and PRN above detectable levels (>5 ELU/mL) at 10 weeks of age compared with controls (192 of 206 [93.2%] vs 98 of 193 [50.8%]; *P* < .001), whether or not the mother had received Tdap within the previous 5 years (Table 2). Similarly, the percentage of infants lacking detectable IgG to either PT and PRN was 19.5% (41 of 210) of those who received the aP vaccine vs 45.0% (90 of 200) of infants in the control group at 6 weeks, decreasing to 0% among the 206 infants who received the aP vaccine vs 11.9% (23 of 193) in the control group at 10 weeks (*P* < .001) (Table 2).

**Table 2.** PT and PRN Antibody Levels Measured at 10 Weeks of Age

Maternal Tdap Status	Pertussis Antibody	No./Total No. (%)		Difference in Proportions, %	OR (95% CI)	*P* Value
		aP Group	Control Group			
Maternal Tdap within 5 y	PT and PRN >5	43/47 (91.5)	27/44 (61.4)	30.1	NA	<.001
No maternal Tdap	PT and PRN >5	149/159 (93.7)	71/149 (47.7)	46.1	NA	<.001
Combined	PT and PRN >5	192/206 (93.2)	98/193 (50.8)	42.4	13.3 (7.2-24.5)	<.001
Maternal Tdap <5 y	PT and PRN <5	0/47	3/44 (6.8)	6.8	NA	<.001
No maternal Tdap	PT and PRN <5	0/159	20/149 (13.4)	13.4	NA	<.001
Combined	PT and PRN <5	0/206	23/193 (11.9)	11.9	Not calculable	<.001

Abbreviations: aP, acellular pertussis; NA, not applicable; OR, odds ratio; PRN, pertactin; PT, pertussis toxin; Tdap, tetanus toxoid, reduced diphtheria toxoid, and pertussis antigen content.

##### Secondary Outcomes

At birth, baseline maternal serum pertussis antibody levels (PT, FHA, and PRN) were similar in both the aP and control groups except for slightly higher FHA antibody levels in the aP group (Table 3). At 6 weeks, GMCs for pertussis IgG antibody were significantly higher in the aP group compared with the control group (PT, 7.46 ELU/mL; 95% CI, 6.58-8.46 ELU/mL vs 4.80 ELU/mL; 95% CI, 4.21-5.47 ELU/mL; *P* < .001; PRN, 10.88 ELU/mL; 95% CI, 8.89-13.32 ELU/mL vs 7.37 ELU/mL; 95% CI, 6.03-9.01 ELU/mL; *P* = .008; and FHA, 35.63 ELU/mL; 95% CI, 31.15-40.76 ELU/mL vs 19.37 ELU/mL; 95% CI, 15.83-23.71 ELU/mL; *P* < .001) (Table 3). At 10 weeks, recipients of aP vaccine at birth had significantly higher GMCs of IgG to PT (4-fold higher), PRN, and FHA, but at 6 and 8 months, only GMCs of IgG to PT and FHA were significantly higher in the aP group than the control group (Table 3).

**Table 3.** Pertussis IgG Antibody (GMC and Detectable) Levels by Study Group and Age

Age at Visit	aP Group^a^		Control Group^b^		*P* Value
	No./Total No. (% Detectable)^c^	GMC, ELU/mL (95% CI)	No./Total No. (% Detectable)^c^	GMC, ELU/mL (95% CI)	
Antibody responses to pertussis toxin					
Maternal	135/216 (62.5)	7.75 (6.74-8.92)	121/217 (55.8)	6.63 (5.78-7.60)	.12
6 wk	136/210 (64.8)	7.46 (6.58-8.46)	74/198 (37.4)	4.80 (4.21-5.47)	<.001
10 wk	199/205 (97.1)	25.42 (22.62-28.56)	112/193 (58.0)	6.04 (5.34-6.83)	<.001
6 mo	201/201 (100)	42.63 (38.79-46.85)	184/185 (99.5)	32.17 (29.48-35.10)	<.001
8 mo	181/181 (100)	52.80 (48.16-57.88)	183/183 (100)	45.18 (41.62-49.04)	.01
Antibody responses to pertactin					
Maternal	131/218 (60.1)	13.28 (10.60-16.64)	131/218 (60.1)	10.12 (8.30-12.34)	.08
6 wk	135/210 (64.3)	10.88 (8.89-13.32)	135/200 (67.5)	7.37 (6.03-9.01)	.008
10 wk	199/205 (97.1)	31.62 (27.22-36.73)	156/193 (80.8)	14.11 (12.00-16.59)	<.001
6 mo	197/201 (98.0)	45.08 (39.50-51.45)	181/185 (97.8)	37.36 (31.79-43.9)	.08
8 mo	179/181 (98.9)	88.62 (78.00-100.68)	181/183 (98.9)	79.62 (70.20-90.32)	.24
Antibody responses to filamentous hemagglutinin					
Maternal	204/216 (94.4)	36.86 (30.86-44.01)	188/211 (89.1)	28.22 (23.40-34.03)	.04
6 wk	209/210 (99.5)	35.63 (31.15-40.76)	157/196 (80.1)	19.37 (15.83-23.71)	<.001
10 wk	205/205 (100)	122.19 (109.43-136.40)	188/192 (97.9)	27.31 (23.94-31.17)	<.001
6 mo	201/201 (100)	191.95 (175.12-210.40)	185/185 (100)	128.25 (115.46-142.45)	<.001
8 mo	181/181 (100)	260.53 (238.74-284.30)	183/183 (100)	216.65 (197.91-237.17)	.004

Abbreviations: aP, acellular pertussis; ELU, enzyme-linked immunosorbent assay units; GMC, geometric mean concentration; Hib, *Haemophilus influenzae* type b.

^a^ The aP group received the aP vaccine and the hepatitis B vaccine at birth, then diphtheria, tetanus, aP, hepatitis B, and Hib antigens at 6 weeks, 4 months, and 6 months of age.

^b^ The control group received the hepatitis B vaccine at birth, then diphtheria, tetanus, aP, hepatitis B, and Hib antigens at 6 weeks, 4 months, and 6 months of age.

^c^ Protocol participants who had blood samples collected for antibody analysis (% detectable = pertussis toxin, pertactin, and filamentous hemagglutinin antibody >5 ELU/mL).

#### Effect of Maternal Receipt of Tdap Within 5 Years of Enrollment

Significantly higher levels of pertussis antibody were found in maternal serum at birth in both the aP vaccine and control groups when receipt of Tdap within 5 years was confirmed. The GMCs for PT were approximately 2.5-fold higher for those in the aP group than those in the control group, and the GMCs for PRN and FHA were approximately 5-fold higher for those in the aP group than those in the control group (eTables 1-3 in Supplement 2).

At 6 weeks of age, levels of pertussis antibody were higher in infants in the aP group compared with those in the control group regardless of maternal prepregnancy Tdap (eTable 1-3 in Supplement 2). By 10 weeks of age, pertussis IgG levels (PT, FHA, and PRN) in infants in the aP group who were born to mothers with no prior Tdap were higher than in infants in the control group who were born to mothers with confirmed Tdap less than 5 years prior (eTables 1-3 in Supplement 2).

Infants who received the aP vaccine at birth, regardless of maternal vaccine status, had significantly higher antibody levels at 24 weeks for PT and FHA compared with infants in the control group, while at 32 weeks, levels were significantly higher only for the specific subgroups of FHA (maternal Tdap <5 years and no Tdap) and PT (no maternal Tdap <5 years) (eTables 1-3 in Supplement 2).

Overall, maternal receipt of Tdap within 5 years of delivery or the presence of detectable maternal antibody at birth resulted in lower postprimary pertussis antibody levels (at 32 weeks of age) among infants in both the aP and control groups. The highest pertussis antibody levels (PT, PRN, and FHA) at 32 weeks were among infants in the aP group who were born to mothers who had not received Tdap within the past 5 years, followed by infants in the control group who were born to mothers who had not received Tdap within the last 5 years (eTables 1-3 in Supplement 2).

#### Antibody Responses to Other Vaccine Antigens

Infants who received the aP vaccine at birth (n = 181 with sera available for testing) had significantly lower GMCs to 4 concomitant antigens (hepatitis B, Hib, tetanus, and diphtheria) at 32 weeks (Table 4). However, despite this finding, the proportion of infants attaining antibody levels above putative protective thresholds was not significantly different between the groups. There were also no significant differences in antibody responses to any pneumococcal vaccine serotype between infants who received the aP vaccine at birth and controls (Table 4).

**Table 4.** Concomitant Antigen Antibody Responses at 8 Months of Age After Completion of Primary Vaccination

Antibody	Threshold	aP Group^a^		Control Group^b^		*P* Value for GMC	*P* Value for Reaching Protective Threshold^d^
		No./Total No. (% >Threshold)^c^	GMC, ELU/mL (95% CI)	No./Total No. (% >Threshold)^c^	GMC, ELU/mL (95% CI)		
Hepatitis B	>10 mIU/mL	149/150 (99.3)	1218 (984-1506)	145/145 (100)	2275 (1883-2747)	<.001	.33
	>100 mIU/mL	143/150 (95.3)		141/145 (97.2)		<.001	.39
Hib	>0.15 μg/mL	176/182 (96.7)	1.53 (1.27-1.85)	177/183 (96.7)	2.12 (1.76-2.57)	.02	.99
	>1 μg/mL	111/182 (61.0)		136/183 (74.3)		.02	.006
Diphtheria	>0.1 IU/mL	180/181 (99.4)	1.24 (1.09-1.41)	183/183 (100)	1.78 (1.57-2.03)	<.001	.31
	>1 IU/mL	106/181 (58.6)		140/183 (76.5)		<.001	<.001
Tetanus	>0.1 IU/mL	181/181 (100)	2.04 (1.84-2.27)	183/183 (100)	2.69 (2.44-2.96)	<.001	
	>1 IU/mL	149/181 (82.3)		170/183 (92.9)		<.001	.002
Pneumococcal serotype							
PnC 1	NC	186	0.93 (0.82-1.06)	179	0.93 (0.82-1.06)	.98	NC
PnC 4	NC	186	1.54 (1.37-1.73)	179	1.51 (1.36-1.68)	.86	NC
PnC 5	NC	186	2.15 (1.91-2.42)	179	1.89 (1.67-2.14)	.14	NC
PnC 6A	NC	186	0.33 (0.27-0.39)	179	0.29 (0.24-0.35)	.36	NC
PnC 6B	NC	186	0.85 (0.75-0.98)	179	0.73 (0.64-0.83)	.10	NC
PnC 7F	NC	186	2.05 (1.83-2.29)	179	1.97 (1.77-2.20)	.62	NC
PnC 9V	NC	186	1.74 (1.53-1.99)	179	1.52 (1.34-1.73)	.15	NC
PnC 14	NC	186	2.46 (2.13-2.84)	179	2.57 (2.23-2.97)	.66	NC
PnC 18C	NC	186	2.63 (2.25-3.08)	179	2.25 (1.93-2.64)	.17	NC
PnC 19A	NC	186	0.27 (0.23-0.32)	179	0.27 (0.23-0.31)	.92	NC
PnC 19F	NC	186	3.25 (2.84-3.73)	179	3.14 (2.76-3.57)	.72	NC
PnC 23F	NC	186	1.02 (0.88-1.18)	179	0.93 (0.81-1.07)	.36	NC

Abbreviations: aP, acellular pertussis; ELU, enzyme-linked immunosorbent assay units; GMC, geometric mean concentration; Hib, *Haemophilus influenzae* type b; NC, not calculated; PnC, pneumococcal serotype.

^a^ The aP group received the aP vaccine and the hepatitis B vaccine at birth, then diphtheria, tetanus, aP, hepatitis B, and Hib antigens at 6 weeks, 4 months, and 6 months of age.

^b^ The control group received the hepatitis B vaccine at birth, then diphtheria, tetanus, aP, hepatitis B, and Hib antigens at 6 weeks, 4 months, and 6 months of age.

^c^ Protocol participants who had blood samples collected for antibody analysis.

^d^ Determined by use of the χ^2^ test.

#### Reactogenicity and Safety

Overall, less than 1% of infants had fever (temperature, ≥38.0°C) within 2 days after the birth dose of aP vaccine (n = 0) and/or HBV (n = 1) (eTable 4 in Supplement 2). Infants given aP vaccine at birth had no increase in local injection site or systemic reactions (eTable 4 in Supplement 2). There was no difference in adverse events after the dose at 32 weeks between groups.

## Discussion

### Immunogenicity of the aP Vaccine at Birth

In the largest study to date, to our knowledge, administration of aP vaccine without diphtheria and tetanus and HBV within 5 days of birth resulted in significantly higher antibodies to pertussis (both PT and PRN) by 10 weeks of age compared with controls receiving only HBV at birth. In addition, receipt of aP vaccine at birth resulted in higher pertussis antibodies (PT, PRN, and FHA) by 6 weeks of age regardless of whether the mother had received Tdap within 5 years of delivery, although this result did not achieve statistical significance. These results indicate that a birth dose of aP vaccine is immunogenic in newborns and significantly narrows the immunity gap between birth and 14 days after receipt of DTaP at 6 or 8 weeks of age, marking the critical period when infants are most vulnerable to severe pertussis infection.

These results confirm and expand on those from 3 earlier small studies that have examined administration of the aP vaccine at birth.^10,11,12^ The 2 most similar studies—a previous pilot study^11^ and a German study^10^—both using aP vaccine produced by the same manufacturer, found statistically significantly higher GMCs of anti-PT, anti-PRN, and anti-FHA IgG antibody at 2 or 3 months of age in infants who received the aP vaccine at birth compared with those who had not been vaccinated.

### Safety of the aP Vaccine at Birth

In our study, receipt of the aP vaccine at birth was found to be safe and well tolerated. More important, in this study, the prevalence of fever after receipt of the birth dose, which can mistakenly be associated with potential sepsis and result in additional investigations in the neonatal period, was similar in both the group that received the aP vaccine at birth and the control group. We found no increased risk of local adverse events after dose 4 of aP-containing vaccines at 32 weeks in infants given the aP vaccine at birth.

### Effect of aP Vaccine at Birth on Pertussis Antibody Responses to Later Doses

In our study, pertussis antibody levels were significantly higher for PT and FHA and were nonsignificantly different for PRN at 32 weeks in recipients of the aP vaccine at birth compared with controls. This finding is similar to results from previous studies using a GlaxoSmithKline-manufactured aP vaccine^10,11^ but contrasts with results from a small study in the United States, in which Sanofi Pasteur–manufactured DTaP administered at birth resulted in lower GMCs for both PT and FHA after primary vaccination than among controls.^9^ These contrasting findings may be related to the different compositions of the pertussis antigens in the GlaxoSmithKline (3 components) and Sanofi Pasteur (5 components) vaccine, an effect of the administration of concomitant diphtheria and tetanus toxoid, or some other factor, but they are consistent with interference from specific vaccines rather than neonatal immunization itself.^20^

### Effect of Maternal Pertussis Antibodies on Infant Vaccine Responses

The presence of maternal pertussis antibodies at birth can negatively affect postprimary responses to pertussis, diphtheria, and diphtheria-related CRM197 conjugate vaccines with a variety of infant immunization schedules and vaccines.^21,22^ A UK study found reduced (or “blunted”) pertussis, diphtheria, and CRM-based conjugate pneumococcal antibody responses in infants born to mothers who were immunized during pregnancy.^22^ In contrast, enhancement of responses to tetanus-related vaccines also appears to be relatively consistent.^22,23^ Maertens et al^24^ found persistent minor blunting 1 month after a fourth vaccine dose (at 15 months of age) for anti-PT antibodies, but the clinical significance of this result is doubtful.

Our study findings are consistent with others showing that detectable maternal antibody at birth is associated with lower pertussis antibody responses after the primary immunization schedule.^25,26^ In our study, this result was seen in both the infants who received the aP vaccine and those in the control group. The clinical significance of reductions in pertussis antibody related to maternal interference will require ongoing clinical evaluation because there are no accepted serologic correlates of protection.^27^

### Interference of Concomitant Antigen Responses

In our study, a birth dose of the aP vaccine resulted in significantly reduced GMCs to the concomitant antigens Hib, diphtheria, and tetanus at 32 weeks; the proportion above the protective threshold was lower but did not reach statistical significance for hepatitis B. Antibody responses to pneumococcal serotypes were consistently, but nonsignificantly, higher in infants who received the aP vaccine at birth compared with controls. Responses to concomitantly administered antigens are not fully understood. One possibility is that of “bystander” interference, speculated to reflect the induction of strong pertussis T-cell responses interfering with the subsequent induction of CD4^+^ helper T cells.^20^ The exact mechanisms for vaccine interference and its apparent preferential induction after neonatal rather than later immunization remain to be elucidated.^28,29^

### Limitations

Our study was an open-label study, and therefore it is possible that parents were more likely to report symptoms after receipt of the aP vaccine. Lack of an established serologic correlate of protection for pertussis makes interpretation of pertussis antibody results problematic, but data from household contact studies support the importance of a lack of detectable antibody to either PT or PRN.^18,19^ Our study enrolled only term neonates, and therefore we are unable to comment on the immunogenicity and safety of the administration of the aP vaccine at birth for premature infants, in whom immune responses may be reduced.

## Conclusions

Our study has shown that a dose of aP vaccine at birth was immunogenic and safe and may induce earlier protection^3^ through generating “active” humoral immunity. There was evidence of a lower pertussis antibody level after completion of the primary vaccine series in infants born to mothers who had received Tdap within the 5 years prior to delivery, as has been shown for higher antibody levels in mothers receiving Tdap during pregnancy.^22,23^ The clinical significance of this finding is not known but is less likely to be important in settings in which aP booster vaccines in the second year of life are routine.

Although receipt of Tdap during pregnancy is the current recommended strategy, administration of the aP vaccine at birth has the potential to reduce the risk of death and severe morbidity from *Bordetella pertussis* infection in the first 2 months of life among infants whose mothers did not receive Tdap during pregnancy. Programs of the administration of HBV at birth are well established in the United States and Australia, but there remain regions where high coverage for maternal vaccination has been challenging. Despite maternal Tdap vaccination now being recommended widely in high-income countries,^24,25,26,30^ there is likely to remain a cohort of infants who do not benefit from this approach either because the mother was not vaccinated or because her infant was born prematurely.^31^

A monovalent (without diphtheria or tetanus) acellular pertussis vaccine containing genetically modified PT and FHA has been shown to be highly immunogenic in adolescent studies^32^ supporting licensure in Thailand, and a study using the baboon model found that a monovalent aP vaccine had equivalent effectiveness to Tdap during pregnancy.^33^ Availability of a monovalent acellular pertussis vaccine has the potential to be valuable in maternal programs in high-income countries (where tetanus and diphtheria boosting is not needed) and would also facilitate the option of neonatal vaccination.

## Notes

Supplement 1. **Trial Protocol**

Supplement 2. **eAppendix.** Supplementary Methods **eTable 1.** Pertussis Toxin IgG Antibody Levels by Maternal Prepregnancy Tdap Vaccine History and Receipt of aP Vaccine at Birth **eTable 2.** Filamentous Haemaglutinin IgG Antibody Levels by Maternal Prepregnancy Tdap Vaccine History and Receipt of aP Vaccine at Birth **eTable 3.** Pertactin IgG Antibody Levels by Maternal Prepregnancy Tdap Vaccine History and Receipt of aP Vaccine at Birth **eTable 4.** Systemic and Local Reactions Within 2 Days After Birth Dose of Acellular Pertussis Vaccine Compared With Controls
