# Supplementary material for: Immunogenicity and Safety of Monovalent Acellular Pertussis Vaccine at Birth: A Randomized Clinical Trial
Source: JAMA Pediatr. 2018 Sep 10;172(11):1045–52. doi: 10.1001/jamapediatrics.2018.2349 (PMC6248137; doi:10.1001/jamapediatrics.2018.2349)
Supplement: Supplement 1. — Trial Protocol [file jamapediatr-172-1045-s001.pdf]

Clinical Study Protocol

Immunogenicity and safety of acellular pertussis vaccine at birth

Protocol Number: BirthPa120609

Investigational Medicinal Product: Acellular Pertussis Vaccine at Birth

Indication: Immunogenicity and safety of acellular pertussis vaccine given at birth in healthy infants

Development Phase: II

Principal Investigator:
Professor Peter McIntyre, Director
National Centre for Immunisation Research and
Surveillance of Vaccine Preventable Diseases

Protocol Version: Amendment 6

Date of Protocol: 9th November 2010

Compliance: This study will be conducted in accordance with the standards of Good Clinical Practice, as defined by the International Conference on Harmonisation, the principles outlined in the Declaration of Helsinki and all applicable Australian regulatory requirements. .

This protocol includes information and data that contain trade secrets and privileged or confidential information. This information must not be made public without written permission from the investigator. These restrictions on disclosure will apply equally to all future information supplied to you. This material may be disclosed to and used by your staff and associates as may be necessary to conduct the clinical study.

Sponsors:

Royal Alexandra Hospital for Children
Corner Hainsworth St and Hawkesbury Rd
Westmead NSW 2145

Murdoch Children's Research Institute
Flemington Road, Parkville Vic. 3052

Women's and Children's Hospital
79 King William Rd.
Adelaide, SA 5006

Princess Margaret Hospital for Children
Roberts Road, Subiaco, Perth, WA 6008

Investigator Signature

Study Title: Immunogenicity and safety of acellular pertussis vaccine at birth

Protocol Number: BirthPa120609

I have read this clinical protocol and confirm that, to the best of my knowledge, it accurately describes the design and conduct of the study title "Immunogenicity and safety of acellular pertussis vaccine at birth"

Professor Peter McIntyre
Director, National Centre for Immunisation Research and Surveillance of Vaccine Preventable Diseases
Professor, University of Sydney
Senior Staff Specialist in Infectious Diseases
The Children's Hospital at Westmead
Locked Bag 4001
Westmead NSW 2145
PeterM@chw.edu.au

Signature Date:

Professor Terry Nolan
Head, Vaccine and Immunisation Research Group
Murdoch Childrens Research Institute
Head, School of Population Health
University of Melbourne
Level 5, 207 Bouverie Street
CARLTON, VIC 3010
t.nolan@unimelb.edu.au

Signature Date:

Dr Helen Marshall
Director
Vaccinology and Immunology Research Trials Unit
Discipline of Paediatrics
Women's and Children's Hospital
72 King William Rd
North Adelaide 5006
Helen.marshall@adelaide.edu.au

Signature Date:

A/Prof Peter Richmond
Associate Professor
Princess Margaret Hospital for Children
GPO Box D184
Perth WA 6540
prichmond@meddent.uwa.edu.au

Signature Date:

Dr Nicholas Wood
Immunisation Research Fellow
National Centre for Immunisation Research and
Surveillance of Vaccine Preventable Diseases
Children's Hospital at Westmead
Locked Bag 4001
Westmead NSW 2145
Nicholw3@chw.edu.au

Signature Date:

Table of content

Sponsors: ... 2
Investigator Signature ... 3
1 General Information ... 8
1.1 Protocol title, protocol identifying number, and date. Any amendment(s) should also bear the amendment number(s) and date(s). ... 8
1.2 Protocol Number: BirthPa120609 ... 8
1.3 Name and address of the sponsors and study monitor (if other than the sponsor). ... 8
1.4 Name and title of the person(s) authorized to sign the protocol and the protocol amendment(s) for the sponsor. ... 8
1.5 Name, title, address, and telephone number(s) of the sponsor's medical expert (or dentist when appropriate) for the trial (site-specific). ... 8
1.6 Name and title of the investigator(s) who is (are) responsible for conducting the trial, and the address and telephone number(s) of the trial site(s). ... 9
1.7 Name, title, address, and telephone number(s) of the qualified physician (or dentist, if applicable), who is responsible for all trial-site related medical (or dental) decisions (if other than investigator). ... 9
2 Background Information ... 10
2.1 Name and description of the investigational product(s). ... 10
2.2 A summary of findings from nonclinical studies that potentially have clinical significance and from clinical trials that are relevant to the trial. ... 10
2.2.1 Disease burden from early infant pertussis: ... 10
2.2.2 Current childhood pertussis vaccine coverage and immunisation schedules: ... 11
2.2.3 Potential strategies for prevention of early infant pertussis: ... 11
a. Maternal immunisation during pregnancy[13]: ... 11
b. Cocooning of infants through parental immunization: ... 11
c. Direct protection of infants through newborn immunisation: ... 11
2.3 Summary of the known and potential risks and benefits, if any, to human subjects.. 11
a. Whole cell pertussis vaccines ... 11
b. Acellular pertussis vaccines ... 12
Table 1: Studies of Pa vaccine at birth ... 12
2.4 Description of and justification for the route of administration, dosage, dosage regimen, and treatment period(s). ... 12
2.4.1 Concomitant antigen responses: ... 12
2.4.2 Influence of maternal antibody: ... 13
2.4.3 Timing and interval of Pa vaccine: ... 13
2.4.4 Cellular immune responses: ... 13
2.4.5 Validity of surrogate measures of protection against pertussis from vaccination: 13
2.5 A statement that the trial will be conducted in compliance with the protocol, GCP as defined by the International Conference on Harmonisation and the applicable Australian regulatory requirement(s). ... 14
2.6 Description of the population to be studied. ... 14
3 Trial Objectives and Purpose ... 14
3.1 Detailed description of the objectives and the purpose of the trial. ... 14
3.2 HYPOTHESES ... 15
4 Study Objectives and Endpoints ... 15
4.1 Primary: ... 15
4.2 Secondary: ... 15
4.3 Measurement of antibody and CMI responses: ... 15

5 Study Design 16
5.1 Study type: 16
5.2 Stratification: 16
5.3 Randomisation: 16
5.4 Maintenance of trial treatment randomization codes and procedures for breaking codes. 17
5.5 Measures taken to minimize/avoid bias, including: 17
5.5.1 Randomization. 17
5.5.2 Blinding. 17
6 Study Population and Procedures 17
6.1 Eligibility Criteria: 18
6.1.1 Subject inclusion criteria: 18
6.1.2 Subject exclusion criteria: 18
6.2 Reasons to delay vaccination 18
6.3 Subject withdrawal criteria (i.e. terminating investigational product treatment/trial treatment) and procedures specifying: 18
6.3.1 When and how to withdraw subjects from the trial/ investigational product treatment. 18
6.3.2 The type and timing of the data to be collected for withdrawn subjects. 19
6.3.3 Whether and how subjects are to be replaced 19
6.3.4 The follow-up for subjects withdrawn from investigational product treatment/trial treatment. 19
6.4 Halting Rules 19
7 Treatment 20
7.1 A description of the trial treatment(s) and the dosage and dosage regimen of the investigational product(s). Also include a description of the dosage form, packaging, and labelling of the investigational product(s). 20
7.2 Accountability procedures for the investigational product(s), including the placebo(s) and comparator(s), if any. 21
8 Schedule of Assessment and study procedure 21
8.1 Active Study 21
8.1.1 Time definition and determination of appointment dates 21
8.1.2 Study period 21
a. Informed Consent 21
b. Pre-Vaccination 22
Table 2: Schedule and groups: 22
8.2 Medication(s)/treatment(s) permitted (including rescue medication) and not permitted before and/or during the trial. 23
8.3 Clinical and Laboratory Procedures 23
8.3.1 Clinical Procedures 23
8.3.2 Blood Samples for Immunogenicity Analysis 24
Table 1: Blood Collection intervals and volume 24
9 Procedures for monitoring subject compliance. 24
10 Adverse Events 24
10.1 Definitions 24
10.1.1 Adverse Event (AE) 24
a. Solicited Adverse Event 25
c. Unsolicited Adverse Event 25
10.1.2 Serious Adverse Event (SAE) 25
10.2 Assessment of Causality 26
10.3 Assessment of Intensity 26

Table 2 Solicited Local AE Definitions and Intensity Grading 27
Table 3 Solicited Systemic AE Definitions and Intensity Grading 27
10.4 Adverse Event Recording 28
10.4.1 Solicited and Unsolicited Adverse Events 28
10.4.2 New Onset of Chronic Illness 28
10.4.3 Serious Adverse Event (SAE) 28
10.5 Serious Adverse Event Reporting 28
10.6 Adverse Event Follow-up 29
10.6.1 The type and duration of the follow-up of subjects after adverse events. 29
10.7 Regulatory Requirements 29
10.8 IEC Reporting Requirements 30
10.9 Composition of DSMB 30
11 Assessment of Efficacy 30
11.1 Specification of the efficacy parameters. 30
11.1.1 Antibodies to pertussis: 30
11.1.2 Antibodies to other vaccine antigens: 30
11.1.3 Cellular immune responses – 30
12 Methods and timing for assessing, recording and analysing of efficacy parameters. 31
13 Administrative Aspects 31
13.1 Initiation of the study 31
13.2 Monitoring of the study 31
13.3 Protocol Deviation / Violations 31
13.4 Confidentiality 32
14 Statistics 32
14.1 A description of the statistical methods to be employed, including timing of any planned interim analysis(ses). 32
14.2 The number of subjects planned to be enrolled. 32
14.2.1 Antibody measures: 32
14.2.2 Cell mediated immunity: 33
14.3 The level of significance to be used. 33
14.4 Statistical criteria for the termination of the trial. 33
14.5 Procedure for accounting for missing, unused, and spurious data. 33
14.6 The selection of subjects to be included in the analyses 33
15 Direct Access to Source Data/Documents 33
16 Quality Control and Quality Assurance 33
17 Ethics 34
18 Data Handling and Record Keeping 34
19 Financing and Insurance 34
20 References 36

PROTOCOL
Amendment 1 Dated: 15 May 2009
Amendment 2 Dated: 12 June 2009
Amendment 3 Dated: 24 November 2009
Amendment 4 Dated: 23rd April 2010
Amendment 5 Dated: 28th July 2010
Amendment 6 Dated: 9th November 2010

1 General Information

1.1 Protocol title, protocol identifying number, and date. Any amendment(s) should also bear the amendment number(s) and date(s).
Immunogenicity and safety of acellular pertussis vaccine at birth
(Short title: Pertussis Vaccine From Birth)

1.2 Protocol Number: BirthPa120609

1.3 Name and address of the sponsors and study monitor (if other than the sponsor).

Royal Alexandra Hospital for Children
Corner Hainsworth St and Hawkesbury Rd
Westmead NSW 2145

Murdoch Childrens Research Institute
Flemington Road, Parkville Vic. 3052

Women's and Children's Hospital
72 King William Rd.
Adelaide, SA 5006

Princess Margaret Hospital for Children.
Roberts Road, Subiaco, Perth, WA 6008

1.4 Name and title of the person(s) authorized to sign the protocol and the protocol amendment(s) for the sponsor.

Principal investigator at each participating centre

1.5 Name, title, address, and telephone number(s) of the sponsor's medical expert (or dentist when appropriate) for the trial (site-specific).

Professor David Isaacs
Department of Infectious Diseases
The Children's Hospital, Westmead
Cnr Hainsworth at and Hawkesbury Rds
Westmead NSW 2145

1.6 Name and title of the investigator(s) who is (are) responsible for conducting the trial, and the address and telephone number(s) of the trial site(s).

Professor Peter McIntyre
Director, National Centre for Immunisation Research and Surveillance of Vaccine Preventable Diseases
Professor, University of Sydney
Senior Staff Specialist in Infectious Diseases
The Children's Hospital at Westmead
Locked Bag 4001
Westmead NSW 2145
PeterM@chw.edu.au

Professor Terry Nolan
Head, Vaccine and Immunisation Research Group
Murdoch Childrens Research Institute
Head, School of Population Health
University of Melbourne
Level 5, 207 Bouverie Street
CARLTON, VIC 3010
t.nolan@unimelb.edu.au

Dr Helen Marshall
Director
Vaccinology and Immunology Research Trials Unit (VIRTU)
Discipline of Paediatrics
Women's and Children's Hospital
72 King William Rd
North Adelaide 5006
Helen.marshall@adelaide.edu.au

Dr Nicholas Wood
Immunisation Research Fellow
National Centre for Immunisation Research and Surveillance of Vaccine Preventable Diseases
Children's Hospital at Westmead
Locked Bag 4001
Westmead NSW 2145
Nicholw3@chw.edu.au

A/Prof Peter Richmond
Associate Professor
Princess Margaret Hospital for Children
GPO Box D184
Perth WA 6540
prichmond@meddent.uwa.edu.au

1.7 Name, title, address, and telephone number(s) of the qualified physician (or dentist, if applicable), who is responsible for all trial-site related medical (or dental) decisions (if other than investigator).

Local Investigator at each participating centre listed above

2 Background Information

2.1 Name and description of the investigational product(s).

Tricomponent acellular pertussis vaccine: contains 25ug pertussis toxoid, 25ug filamentous haemagglutinin, 8ug pertactin, 0.5mg aluminium salt.

2.2 A summary of findings from nonclinical studies that potentially have clinical significance and from clinical trials that are relevant to the trial.

2.2.1 Disease burden from early infant pertussis:

In the past decade, there has been a resurgence of pertussis in Australia and other developed countries with long-established immunisation programs, in those over the age of 10 years (waning immunity) and infants under the age of 5 months (too young for full immunization).[1-3] Unimmunised infants are most likely to develop severe disease requiring hospitalization, frequently complicated by apnoea, seizures, encephalopathy or death.[3,4] Available data on pertussis in Australia include notifications to public health, hospitalizations and deaths. [1] Pertussis notification rates in infants aged less 6 months remain higher than any other age group, despite maximal pertussis vaccine coverage in older children [1] Infant hospitalisations average 400 per year, more than 80% before 5 months of age. (Figure 1) Five months is the earliest the two doses needed for significant protection [5,6] can be reliably delivered under current immunisation schedules. Approximately 35% of hospital admissions occur before 8 weeks of age (Figure 1) Of the 18 deaths coded as due to pertussis in Australia 1993-2004, all but two were less than 6 months of age.[1] Death certificate records are thought to underestimate infant deaths from pertussis by a factor of at least 3.[6]

Figure 1: Pertussis hospitalisations under 12 months of age 1994-2004 (n=4114) [8]

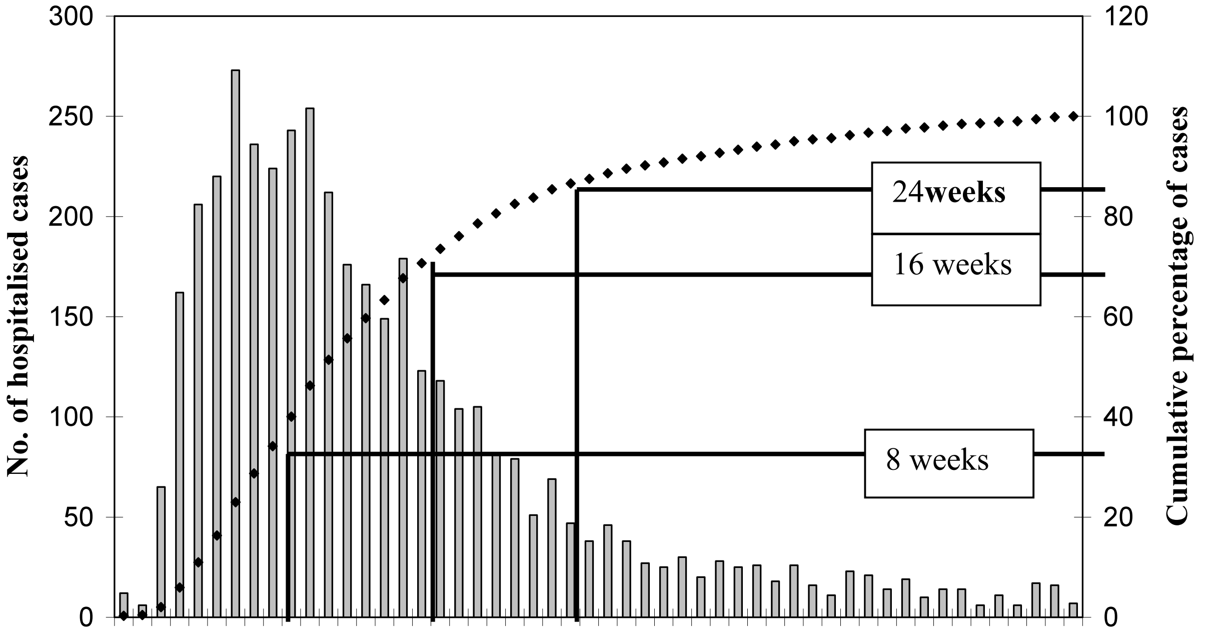

Worldwide, some 300 000 children under 5 years old die each year from pertussis.[7] This same pattern of pertussis affecting infants who are too young to have received vaccination is also seen in other countries with high levels of immunisation, such as Germany[5] and the UK.[6] In countries such as Australia with high immunisation coverage in children[4]adults, particularly parents, are the most important source of infection for infants,

2.2.2 Current childhood pertussis vaccine coverage and immunisation schedules:

Pertussis immunisation schedules differ significantly around the world but currently do not start earlier than 6 weeks of age. The World Health Organisation (WHO) recommends a schedule 6, 10, 14 weeks for primary immunisation against pertussis and a 2,3,4 month schedule is used in many European countries.[7, 10] Australia follows the US schedule of primary immunisation at 2, 4 and 6 months. The Australian Childhood Immunisation register (ACIR) records that by the age of 12 months, 92% of children have received 3 doses, but successive doses are more likely to be delayed, especially in Indigenous infants.[9] Accelerated schedules reduce delay in subsequent doses.[9] The first dose of pertussis-containing vaccine is licensed for use from 6 weeks of age and is accepted as a valid dose by the ACIR when given at 6 weeks.

2.2.3 Potential strategies for prevention of early infant pertussis:

Strategies to reduce early infant pertussis have been summarised[11,12] and are discussed below.

Maternal immunisation during pregnancy[13]:

Maternal vaccination has been extremely successful in preventing neonatal tetanus in developing communities, but for pertussis has significant hurdles. First, licensure of any therapeutic substance in pregnancy is difficult and even when recommended often has low uptake, such as seen with maternal influenza vaccination. Second, although there is some suggestion that passively transferred maternal antibody provides some protection, this is not universal and declines rapidly after birth.[14] Third, maternal levels wane rapidly, so re-vaccination may be necessary. Studies of maternal Pa vaccination are in progress, supported by the National Institutes of Health in the US and by industry sponsors in Canada.

Cocooning of infants through parental immunization:

Parents are the single most common identified source of pertussis transmission to infants, though a source is often not known.[4] In Australia[15], USA, France, Austria and Germany it is recommended that all persons who come into contact with newborns receive a dTpa booster shortly after delivery – a "cocoon" strategy. High vaccination coverage is difficult to achieve unless funding and distribution is directly supported, which is not currently the case in Australia or elsewhere.

Direct protection of infants through newborn immunisation:

The desirability of vaccination at the youngest possible age was recognised in the 1940s because of high mortality among neonates. Successful neonatal vaccination must result in the early protective immunity, be well tolerated and safe. Only BCG and Hepatitis B vaccines are routinely administered at birth.[8]

2.3 Summary of the known and potential risks and benefits, if any, to human subjects.

a. Whole cell pertussis vaccines

Vaccination against pertussis at birth with whole cell pertussis (Pw) was studied from the 1940s, but questions about immune tolerance discouraged later studies[16] Although the study which raised concerns that Pw vaccine in the first week of life might be followed by immune tolerance was small and poorly designed [17] it was influential in discouraging further pursuit

of early vaccine schedules. However, Galaska and Halsey's review in 1984 concluded that the overall evidence for tolerance was weak.[16] Early studies of Pw vaccines measured only agglutinins, had small numbers of subjects and often did not give data on responses prior to dose 3. [16] A more recent study, comparing a 2,3,4 month DTPw schedule with 3,5,9 months, found that while D and T antibody titres were lower for the accelerated schedule, PT was non-significantly higher.[18] The conclusion that tolerance following early DTPw was not a major problem was also supported by a US study.[19] Unlike Pw, the response to acellular pertussis (Pa) vaccine seems less likely to be interfered with by maternal antibody;[14]Pa has replaced Pw in most rich countries .

Acellular pertussis vaccines

As trials to measure disease endpoints in neonates are unfeasibly large, all studies of Pa-containing vaccines given at birth must rely on antibody responses as surrogate markers of protection. Three small studies have recently been completed in Italy[20] Germany[21], and the USA.[22]The studies differ in several ways, including the vaccine and schedule used (Table 1). Our pilot study (CHW ethics approval 2003/07) conducted from 2005 to 2007 included an arm with 2 Pa doses before 8 weeks. This demonstrated significantly higher antibody by 8 weeks in the 2-dose group.[23] (Manuscript attached)

Table 1: Studies of Pa vaccine at birth

	**Italy[20]**	**USA[21]**	**Germany[22]**	**Australia[23]**
**Sample size**	n= 91	n= 50	n=110	n=76
**Birth vaccine used**	DTPa	DTPa	Pa	Pa
**Manufacturer**	Chiron	Sanofi Aventis	GSK	GSK
**Schedule**	3, 5, 11 months	2, 4, 6 months	2, 4, 6 months	1, 2, 4, 6 months
**Antibody post dose 2 (2 to 3m)**	PT only	Not measured	PT, PRN, sig higher	PT, PRN, sig higher
**Reduced antibody @ 7 months**	yes	yes	no	no

The pilot study results, in the context of disease burden timing above, have established that 2 doses prior to 8 weeks is likely to be beneficial and set the scene for a more definitive study examining a readily implementable accelerated schedule to achieve this.

2.4 Description of and justification for the route of administration, dosage, dosage regimen and treatment period(s).

Rationale For Study Design And Key Questions From Our Pilot Study:

2.4.1 Concomitant antigen responses:

Other vaccine antigens co-administered with Pa-containing vaccines include diphtheria, tetanus, polio, Hepatitis B and *Haemophilus influenzae* type b (Hib). In the German study, attainment of Hib antibody responses indicating protection (>0.15ug/ml) was significantly lower after the first 3 doses (88% vs. 98%).[22] Reduced Hib immunogenicity has been associated with DTPa-Hib combination vaccines, but has only emerged as a clinical problem in the UK prior to that country introducing a Hib booster.[24] In Australia, a Hib booster has always been routine.[15]

There was no significant difference in response to other antigens. In our pilot study, hepatitis B antibody levels were non-significantly lower in the birth Pa group, but all participants were above the accepted threshold for protection (>10mIU/L). This study will examine IgG responses to D, T, Hib and hepatitis B post primary immunisation.

2.4.2 Influence of maternal antibody:

The effect of pre-existing maternal pertussis Abs, particularly higher levels resulting from maternal immunization, on responses to Pa vaccines at birth or subsequently is uncertain.[14]Our pilot data suggest that two doses of Pa vaccine (at birth and 1-2 months old) are immunogenic in the presence of detectable maternal antibody, but did not include immunised mothers. Since 2007, NSW Health has required that all healthcare workers with patient contact be given dTpa booster vaccination and non-mandatory guidelines are in place in other jurisdictions. As healthcare workers participate more commonly in vaccine trials, this should facilitate recruitment to a study arm with mothers immunised with dTpa. Irrespective of the results of maternal immunisation studies, the question of persistence of adequate antibody in subsequent pregnancies remains.

2.4.3 Timing and interval of Pa vaccine:

Our study suggests that two doses of Pa vaccine, the first as soon as possible after birth and the second separated by a minimum of 4 weeks, give optimal PT IgG levels.[23] Observational studies suggest some level of protection even from one dose.[5] In Germany, protective efficacy against infant hospitalisation was 68% after the first and >90 % after the second dose of DTPa.[5] In Sweden, the incidence of pertussis fell from 230-235 (cases per 100 000 person years) after zero or one dose of pertussis vaccine to 52 after two doses.[25] Pilot data from both the Australian and German birth Pa vaccine trials showed significant rises in antibody after the second dose, at 4 and 8 weeks of age respectively.[22,23] A second dose at 6 weeks of age should be similar, but is more feasible and practical, as current combination vaccines including Pa are licensed from this age and considered a valid dose by the ACIR from 6 weeks. A second dose at 6 weeks of age aligns with routine well baby checks, reducing delivery costs, and is consistent with the current WHO schedule.

2.4.4 Cellular immune responses:

Our pilot study showed that infants who received Pa vaccine at birth had a significant increase in IL5 and IL13 cytokine (TH2) responses to pertussis vaccine antigens at 7 months compared to those vaccinated later. There were no differences in IFNγ and cytokine (TH1) responses to other vaccine antigens between groups.[26] This suggests that birth Pa vaccine results in a TH2 bias, restricted to pertussis responses, compared to controls and raises the question of altered responses following subsequent natural pertussis exposure. Detailed profiling of infant T-cell immunity in response to neonatal pertussis vaccination is thus an essential component of this study.

2.4.5 Validity of surrogate measures of protection against pertussis from vaccination:

Evidence to support the validity of surrogate immunologic measures comes from animal studies[27] and vaccine trials. These trials were conducted in countries with a high incidence of disease (Italy, Germany, Sweden) not using the Pw vaccines then available.[28]The composition of currently available Pa vaccines was determined by detailed immunogenicity studies under the auspices of the US National Institutes of Health.[29] Despite the limited evidence for association between specific levels of individual antibodies and protection against disease, there has been a pragmatic need to measure antibody markers of immune response to Pa vaccines. In the National Institutes of Health (NIH) sponsored comparative immunogenicity trials for acellular pertussis vaccines, seroconversion to PT and PRN measured by ELISA

was defined as a 4x increase in pre-immunisation Elisa Unit (EU) value, to at least 4x the minimum detectable level.[29] The minimum detectable levels were 2 EU for PT and PRN.

All acellular vaccines include **pertussis toxin (PT)** as it is only present in *Bordetella pertussis*, and appears to be the most important single factor in protection against severe disease.[28,30] All current commercially available vaccines also contain **pertactin (PRN)**, an attachment factor protective in the mouse respiratory challenge model.[27] Human data on the correlation between antibody and protection come from household contact studies including vaccine trial participants, allowing pertussis following known exposure to be correlated with post-vaccination antibody.[27-29] The Swedish study found a statistically significant efficacy of 75% (95% CI 0-96%) against typical pertussis when measurable antibody levels to both PT and PRN were present post primary vaccination.[31] The German study found protection significantly correlated only with PRN.[32] Despite limitations in the evidence for specific antibody thresholds and protection against disease, these measures are deemed sufficient for global licensure by all regulatory bodies and the overwhelming body of evidence suggests antibody to both PT and PRN has a role in protection. Accordingly, we have adopted a dichotomous classification, with measurable IgG to both PT and PRN deemed to indicate at least short-term protection against severe disease.

2.5 A statement that the trial will be conducted in compliance with the protocol, GCP as defined by the International Conference on Harmonisation and the applicable Australian regulatory requirement(s).

This trial will be conducted in compliance with the protocol, GCP as defined by the International Conference on Harmonisation and the applicable Australian regulatory requirements.

2.6 Description of the population to be studied.

Healthy newborns, greater than 36 completed weeks gestation, will be randomized to receive acellular pertussis vaccine and hepatitis B vaccine at birth (within 120 hours of birth) or hepatitis B vaccine alone.

3 Trial Objectives and Purpose

3.1 Detailed description of the objectives and the purpose of the trial.

Currently, vaccines to protect against pertussis (whooping cough) are given from 2 months of age, but almost one third of infant hospitalisations for pertussis occur prior to 2 months. Some early studies of older pertussis vaccines suggested that vaccination at birth was not appropriate. However, these studies were small and their results are no longer relevant in the acellular pertussis vaccine era. Three small recently published studies of acellular pertussis vaccine at birth suggest that this has advantages.

This study aims to randomly assign a group of newborn infants to birth acellular pertussis (Pa) vaccine versus current standard practice. Infants will either receive a Pa-containing vaccine at birth and then 6 weeks, four and six months of age **or** the standard schedule with the first dose given at 6 rather than 8 weeks. Antibody responses in the blood, which are believed to correlate with protection, will be compared at 6 weeks, 10 weeks, 6 months and 8 months of age. This study aims to show whether earlier vaccination gives better protection from pertussis at the time when babies are most likely to die from this infection.

3.2 HYPOTHESES

1. IgG antibody responses to pertussis toxin (PT) and pertactin (PRN) in infants receiving monovalent acellular pertussis vaccine (Pa) at birth and routine vaccines at 6 weeks are significantly higher than in unvaccinated infants by 10 weeks of age**,** and remain higher at 6 and 8 months of age
2. Cell-mediated immune responses to PT and PRN at 10 weeks and 8 months of age in infants receiving monovalent acellular pertussis vaccine at birth and routine vaccines at 6 weeks do not differ significantly from those in infants with first doses at 6 weeks.
3. IgG antibody responses to **PT and PRN** at each time point in infants of mothers vaccinated with dTpa within the previous 5 years do not differ significantly from infants of unvaccinated mothers.
4. In infants receiving Pa at birth and routine vaccines at 6 weeks, the prevalence of antibody levels consistent with protection to **other vaccine antigens** is not significantly reduced
5. The frequency, duration and intensity of solicited symptoms in the 6 days following vaccination and unsolicited symptoms until the next vaccination visit (up to 8 months old) will not differ significantly between the infants receiving Pa vaccine at birth and controls.

4 Study Objectives and Endpoints

4.1 Primary

To establish if earlier protection from severe disease due to *Bordetella pertussis*, as measured by PT and PRN antibody (Ab) and cell mediated immune (CMI) responses, is achieved by monovalent acellular pertussis vaccine (Pa) at birth *versus* vaccination schedules commencing at 6 weeks of age.

4.2 Secondary

1. To determine whether maternal receipt of adult-formulated acellular pertussis vaccine (dTpa) within the previous 5 years influences infant Ab or CMI responses.
2. To evaluate interactions with vaccine antigens given concurrently at birth (hepatitis B) or later
3. To evaluate adverse clinical or immunological effects from early use of Pa vaccine.

4.3 Measurement of antibody and CMI responses

Measurement of antibody by a reference laboratory with rigorous standardisation and evidence of reproducibility is essential.[33] The GlaxoSmithKline (GSK) laboratories in Belgium have long experience with pertussis antibody assays, validated by the US Food and Drug Administration, for many clinical trials in infants from 2 months of age. To maximise comparability of the proposed trial with the previous pilot results and to ensure high assay standards, we have contracted with the GSK laboratory to undertake the assays for pertussis antigens, Diphtheria (D), Tetanus (T), hep B, pneumococcal serotypes (10 serotypes if volume of blood sample permits) and Hib. The expertise of this laboratory also enables measurement of antibody to the widest possible range of relevant vaccine antigens using minimal blood volumes. With respect to measurement of CMI, the laboratory of Dr Hollams and Professor Pat Holt, ICHR, Perth has an international reputation and previous experience in pertussis-specific assays and will conduct these assays. In the pilot study, we demonstrated that the transport of viable samples for CMI assay to Perth was possible.

5 Study Design

5.1 Study type

The study will be stratified, randomised but not blinded. Lack of blinding limits the potential to examine non-specific adverse reactions, such as irritability and abnormal crying, but we judge a placebo not justified according to guidelines or the Declaration of Helsinki 1999. The trial will be registered with the Australian Clinical Trials Registry.

5.2 Stratification

This will be by maternal immunisation status / past history of pertussis infection before randomization in two groups:
Stratum A – maternal dTpa vaccine or history of maternal pertussis infection within last 5 years.
Stratum B – no previous maternal dTpa vaccine or dTpa vaccine > 5 years pre-delivery and no history of maternal pertussis infection during last five years.
A maternal history of Pertussis infection will be accepted as positive if the participant's mother reports that the diagnosis was made by blood test or throat swab and confirmation of diagnosis via blood test or throat swab is received by her healthcare provider.
NOTE: If a mother receives dTpa vaccine after delivery of child but before enrolment of infant then infant should be stratified into stratum B.

5.3 Randomisation

Once written informed consent has been provided and eligibility has been confirmed, the participant will be issued with a unique participant number. The participant number will be used to identify the participant for the duration of the study. Neonates will be randomly assigned, in a 1:1 ratio to one of two groups (Table 2):

Birth group (early vaccination) Pa vaccine at birth, 6 weeks, 4 months and 6 months.
Control group (standard vaccination) usual vaccinations at 6 weeks, 4 months and 6 months.

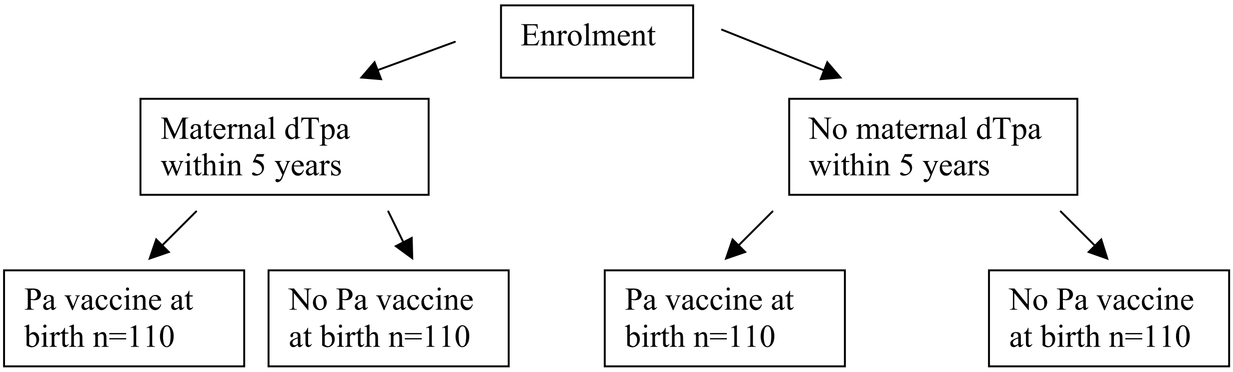

Participants will be randomized using the central randomisation service at the NMHRC Clinical Trial Centre, Sydney, by automated interactive voice response system (IVRS) (available 24hrs). Investigators and study research staff will follow the voice prompts on the IVRS and complete the randomisation worksheet. Participants will be allocated to either the Pa vaccine at birth or No Pa vaccine given at birth groups via the IVRS.

Before randomisation, stratification into either the 'maternal dTpa vaccine prior to birth within 5 years' group / history of maternal pertussis infection < 5years **or** no maternal dTpa

vaccine < 5years / no history of maternal pertussis infection < 5years will be conducted according to maternal verbal report.

Subsequently attempts will be made to confirm maternal verbal report of receipt of dTpa vaccine or history of laboratory confirmed maternal infection by obtaining verification from the maternal healthcare provider. If no confirmation of mother having received dTpa vaccine within last 5 years (prior to enrolment) or history of laboratory confirmed maternal infection with pertussis is obtained, the mother/infant will be re-allocated to the no maternal dTpa vaccine group for the purpose of secondary analysis only.

5.4 Maintenance of trial treatment randomisation codes and procedures for breaking codes.

This study will be unblinded.

5.5 Measures taken to minimize/avoid bias, including:

5.5.1 Randomization.

Participants will be randomized using the central randomisation service at the NMHRC Clinical Trial Centre, Sydney.

5.5.2 Blinding.

The study will be open-label. Although lack of blinding limits the ability of the study to examine the incidence of non-specific adverse reactions, such as irritability and abnormal crying, to do so would require use of a placebo which we judge not to be justified according to current guidelines for trial conduct (Declaration of Helsinki 1999).

6 Study Population and Procedures

This study can fulfil its objectives only if appropriate participants are enrolled. The following eligibility criteria are designed to select participants for whom protocol treatment is considered appropriate. All relevant medical and non-medical conditions should be taken into consideration when deciding whether this protocol is suitable for a particular participant.

Participant eligibility should be reviewed and documented by an appropriately qualified member of the investigator's study team before participants are included in the study.

Recruitment of women may be antenatal or postnatal but randomisation and vaccination (if required) must be within 120 hours of birth. Women indicating interest in the study will be provided with detailed information in writing and orally and will have opportunity to discuss the study with the researchers and family members, their medical advisers or other persons of their choosing. Parents will be informed that participation is voluntary and they may withdraw from the study at any time.

6.1 Eligibility Criteria:

6.1.1 Subject inclusion criteria:

English-speaking women in the last trimester of pregnancy and/or who have recently given birth to a healthy infant will be approached at selected maternity hospitals or Obstetric Practices in the four study sites, Sydney, Adelaide, Melbourne and Perth.

Eligible infants must be healthy (by history and physical examination) at the time of first vaccine, born at 36 completed weeks gestation or greater and less than 120 hours old, whose parents give written informed consent.

6.1.2 Subject exclusion criteria:

- Contraindications to vaccination as listed in the NHMRC Australian Immunisation Handbook 9th Edition. Monovalent acellular pertussis vaccine should not be administered to individuals known to be hypersensitive to any component of the vaccine or residues carried over from manufacture (such as formaldehyde and glutaraldehyde). Synflorix should not be administered to subjects with known hypersensitivity to any component of the vaccine.
- Infant of mother known to be a carrier of hepatitis B virus
- Administration of immunoglobulins and any blood products preceding the first dose of study vaccine or planned administration during the study period.
- Any confirmed or suspected immunosuppressive or immunodeficient condition, in child
- Major congenital defects or serious chronic illness
- Neurologic disease or seizure

6.2 Reasons to delay vaccination

6.2.1 Acute disease at the time of vaccination, which may include conditions, which in the opinion of the treating physician warrant investigation and/or treatment with fever defined as axillary temperature >37.2°C. If the condition resolves before 120 hours from the time of birth, participation may continue.

6.2.2 Vaccination with HBVaxII Paediatric® in the birthing suite before a visit by study staff for the purpose of enrolment is not an exclusion criterion.

6.3 Subject withdrawal criteria (i.e. terminating investigational product treatment/trial treatment) and procedures specifying:

6.3.1 When and how to withdraw subjects from the trial/ investigational product treatment.
Participants may withdraw from the study at any time at the request of their parent / guardian, or they may be withdrawn at any time at the discretion of the Investigator for safety, behavioural or administrative reasons.

If a subject develops any of the following then he/she will be withdrawn from the trial

- Contraindications to vaccination as listed in the NHMRC Australian Immunisation Handbook 9th Edition.
- Serious chronic illness following discussion between investigators at all sites

This will be checked at each visit. If any become applicable during the relevant study period, the subject will not be required to continue the study.

Similarly, the data will not be eliminated from analysis (intention to treat approach) if any of the following occur, however in this case the subject will continue in the study:

- Use of any investigational or non-registered drug or vaccine during the study period.
- Administration of a vaccine not foreseen by the study protocol during the period starting from 30 days before any dose of vaccine(s) and ending 30 days after.
- Administration of immunoglobulins and/or any blood products during the study period.
- Administration of immunosuppressants or other immune-modifying drugs during the study period.
- Diagnosis or suspicion of an immunosuppressive condition during the study period.

6.3.2 The type and timing of the data to be collected for withdrawn subjects.

From an analysis perspective, a 'drop-out' will be any subject who did not come back for the concluding visit foreseen in the protocol. A subject who returns for the concluding visit foreseen in the protocol (i.e. at month 12, 6 months after the full vaccination course) is considered to have completed the study. The reason(s) for dropout shall be recorded. Classifications for dropouts: serious adverse event; non-serious adverse event; protocol violation; consent withdrawal; migrated/moved from the study area; lost to follow-up.

6.3.3 Whether and how subjects are to be replaced.

Participants who withdraw from the study will not be replaced.

6.3.4 The follow-up for subjects withdrawn from investigational product treatment/trial treatment.

Attempts will be made to contact, via phone, email or mail as appropriate, those parents of subjects who do not return for scheduled study visits. Information will be gathered on the subject’s adverse experience, hospitalisation and whether the subject developed any significant medical problem. The information collected should be described on Medication and Adverse event pages and on the Study Conclusion page of the case record form.

6.4 Halting Rules

A DSMB will be established to monitor the safety of the study.

All serious adverse events (SAEs) causally related to the administration of monovalent acellular pertussis vaccine, will be reviewed by the DSMB within 24hrs of notification of the event to the DSMB Chairperson by the Principal Investigator.

The DSMB will make a recommendation regarding continuation of the trial. Enrolment and further dosing of the monovalent acellular pertussis vaccine will be halted pending the outcome of the DSMB review/recommendation.

All other SAEs will be reviewed periodically by the DSMB in accordance with the DSMB Charter.

7 Treatment

7.1 A description of the trial treatment(s) and the dosage and dosage regimen of the investigational product(s).
Also, include a description of the dosage form, packaging, and labelling of the investigational product(s).

Tricomponent acellular pertussis vaccine contains 25µg pertussis toxoid, 25µg filamentous haemagglutinin, 8µg pertactin, 0.5mg aluminium salt per 0.5mls the vaccine is preservative free and presented as a single vial for intramuscular administration. The vaccine will be labelled in accordance with the TGA labelling requirements for clinical trial material.

All vaccinations (routine infant schedule or study related) due will be administered by the study team, using the special batch of Pa vaccine manufactured by GSK Biologicals and commercially available batches of the other vaccines and standard vaccination procedures**.**

The pneumococcal conjugate vaccine used in this study will be Synflorix, manufactured by GSK and containing 10 pneumococcal serotypes with protein D a conserved protein from non-typable Haemophilus influenzae. Synflorix is approved by the Therapeutic Goods Administration for active immunisation of infants and children from the age of six weeks up to two years against disease caused by *Streptococcus pneumoniae* serotypes 1, 4, 5, 6B, 7F, 9V, 14, 18C, 19F and 23F including invasive disease (meningitis, septicaemia and bacteraemic pneumonia) and acute otitis media. (See TGA document attached). This vaccine has equivalent immunogenicity and a comparable safety profile to Prevenar (Wyeth Australia) with the added benefit of wider pneumococcal serotype coverage (serotypes 1, 5, 7F) and non-typable Haemophilus influenzae. Further details are included in the product information attached.

Synflorix will be used instead of Prevenar, which is currently the most widely used pneumococcal conjugate vaccine in Australia.

Other vaccines administered (included on National Immunisation Program)

Infanrix Hexa will be administered, by intramuscular injection, to all infants at 6 weeks, 4 and 6 months old. Infanrix hexa is a DTPa-HBV-IPV-Hib vaccine containing 30 IU diphtheria toxoid, 40 IU tetanus toxoid, 25 ug pertussis toxoid, 25 ug filamentous haemaglutinin, 8 ug pertactin, 10 ug recombinant hepatitis B surface antigen, 40 D-antigen units inactivate poliovirus type 1, 8 D-antigen units type 2, 32 D-antigen units type 3 absorbed onto aluminium hydroxide/phosphate and a vial containing 10ug Hib polysaccharide conjugated to 20-40 ug tetanus toxoid).

A rotavirus vaccine, Rotarix or Rotateq, will be administered orally to all infants according to the standard vaccine schedule in each investigator site.

Rotarix – GlaxoSmithKline (live attenuated RIX4414 human rotavirus strain expressing G1P1[8] outer capsid proteins). Each 1.0 mL monodose of the reconstituted vaccine contains not less than $10^{6.0}$ $CCID_{50}$ (cell culture infectious dose 50%) of the RIX4414 strain; sucrose; dextran 40; sorbitol; amino acids; Dulbecco's Modified Eagle Medium; calcium carbonate; xanthan gum. Calcium carbonate buffer solvent (diluent) supplied for reconstitution.

RotaTeq – CSL Biotherapies/Merck &Co Inc (live, oral pentavalent vaccine). Each 2.0 mL monodose pre-filled dosing tube contains rotavirus reassortants G1, G2, G3, G4 and P1[8]

each with a minimum dose level of at least 2.0 x 10^6 infectious units; sucrose; sodium citrate; sodium phosphate monobasic monohydrate; sodium hydroxide; polysorbate 80; cell culture media; trace amounts of foetal bovine serum.

HB-Vax-II (paediatric) – CSL Biotherapies preservative free vaccine – each 0.5ml dose contains hepatitis B surface antigen protein 5ug adsorbed onto 0.25mg aluminium hydroxide.

Dosage and Administration
The date and batch numbers of vaccines administered at birth, 6 weeks, 4 and 6 months old will be recorded in the Child's Personal Health Record and the Australian Childhood Immunisation Record will be updated.

7.2 Accountability procedures for the investigational product(s), including the placebo(s) and comparator(s), if any.

There is no placebo vaccine in this study. GSK is responsible for the production and shipment of monovalent acellular pertussis vaccine to local study sites. The Investigator at each participating centre is responsible for ensuring the maintenance and viability of the study vaccines and their appropriate storage.

The monovalent pertussis vaccine and Synflorix will be shipped to the investigational study site prior to study start and will be stored under temperature-controlled and monitored conditions (2°C to 8°C) in a secure area. The Study Vaccines must not be frozen.

The Investigator/delegate administering the Study Vaccine must record the dispensing details on the Study Vaccine Administration Form. Used monovalent pertussis vaccine vials and cartons must be retained at study site for product accountability purposes. Any unused monovalent pertussis vaccine will be returned to GSK at the end of the study or destroyed at the study site.

Used syringes are to be disposed of in appropriate sharps containers.

8 Schedule of Assessment and study procedure

8.1 Active Study
The expected duration of subject participation, and a description of the sequence and duration of all trial periods, including follow-up, if any.

8.1.1 Time definition and determination of appointment dates

In general, appointments for study visits which may occur at the study site or in participants' homes, will be determined by the age of each infant. The intervals at which study visits should occur are listed in the second column of the following table. However, there are 'critical intervals' which must be the primary determinant of appointment dates in cases where following the age-related appointment dates would compromise the critical intervals. Appointments forced 'out of age' by the critical intervals can be late but are not permitted to be early.

8.1.2 Study period

a. <u>Informed Consent</u>

Written informed consent must be provided by the parent(s) or guardian(s) before any study-related procedures are performed.

b. Pre-Vaccination

The following assessments will be performed by the Investigator / delegate:

- A review of the maternal medical history, including demographics including name, contact number, address, race. Also any concomitant medication use and vaccination history, pregnancy history (gestational age), maternal pertussis vaccination/infection history and the contact details of the family doctor.
- Infant demographics including name, birth weight, height, head circumference, gestation and infant medical illnesses.
- A brief medical evaluation and other physical examination if clinically indicated.
- Measurement of the participant's axillary temperature.

All relevant data must be recorded in appropriate source documents.

Infants will be followed until 12 months of age when there will be a follow up phone call.

All participants will receive the 4th dose of Synflorix at 12 months of age. This will be arranged by each study investigator/ team at local study sites and may include either a scheduled visit from study staff or administration of Synflorix vaccine by local healthcare / council clinic vaccine provider.

Table 2: Schedule and groups:

(1) Birth dose + 6 weeks, 4, 6 months

(2) Control group - standard schedule 6 weeks, 4, 6 months

Pa regimen	Age	**Group 1**<br>**0, 6 weeks 4, 6 months**	**Group 0**<br>**6 weeks, 4, 6 months**
Subjects		220	220
**Visit no.**			
**1**	**Birth**<br>(Birth to <120 hours old)	**Maternal blood sample***<br>Pertussis antibodies<br>**Vaccine administration**<br>$HepB_1$ aP_1<br>**Observations**<br>Reactogenicity $_1$<br>**Parental vaccine**<br>dTpa (Boostrix) booster	**Maternal blood sample***<br>Pertussis antibodies<br>**Vaccine administration**<br>$HepB_1$<br>**Observations**<br>Reactogenicity<br>**Parental vaccine**<br>dTpa (Boostrix) booster
**2**	**6 weeks**<br>(42 56 days)	**Blood sample** (6 weeks post dose 1)<br>Pertussis antibodies<br>**Vaccine administration**<br>$D_1T_1 Pa_2$-$HepB_2$-IPV_1-PRP-T_1<br>Rotavirus vaccine<br>Synflorix<br>**Observations**<br>Reactogenicity	**Blood sample**<br>Pertussis antibodies<br>**Vaccine administration**<br>$D_1T_1Pa_1$-$HepB_2$-IPV_1-PRP-T_1<br>Rotavirus vaccine<br>Synflorix<br>**Observations**<br>Reactogenicity

3	**10 weeks**	**Blood sample** (28-35 days post dose 2)<br>Pertussis antibodies<br>Cell medicated immunity (subset only)	**Blood sample** (28-35 days post dose 1)<br>Pertussis antibodies<br>Cell mediated immunity (subset only)
4	**4 months**<br>(116-137 days)	**Vaccine administration**<br>$D_2T_2Pa_3$-$HepB_3$-IPV_2-PRP-T_2<br>Rotavirus vaccine<br>Synflorix<br>**Observations**<br>Reactogenicity	**Vaccine administration**<br>$D_2T_2Pa_2$-$HepB_3$-IPV_2 -PRP-T_2<br>Rotavirus vaccine<br>Synflorix<br>**Observations**<br>Reactogenicity
5	**6 months**<br>(176-211 days)	**Blood sample** (2 months post dose 3)<br>Pertussis antibodies<br>**Vaccine administration**<br>$D_3T_3Pa_4$-$HepB_4$-IPV_3-PRP-T_3<br>Synflorix<br>**Observations**<br>Reactogenicity	**Blood sample** (2 months post dose 2)<br>Pertussis antibodies<br>**Vaccine administration**<br>$D_3T_3Pa_3$-$HepB_4$-IPV_3-PRP-T_3<br>Synflorix<br>**Observations**<br>Reactogenicity
6	**8 months**	**Critical interval**<br>60-74 days from visit 5<br>**Blood sample** (2 months post dose 4)<br>Pertussis antibodies<br>Concomitant antigen antibodies – hepatitis B, Hib, polio, diphtheria, tetanus, pneumococcal[1]<br>Cell mediated immunity (subset of participants)	**Critical interval**<br>60-74 days from visit 5<br>**Blood sample** (2 months post dose 3)<br>Pertussis antibodies<br>Concomitant antigen antibodies – hepatitis B, Hib, polio, diphtheria, tetanus, pneumococcal[1]<br>Cell mediated immunity (subset of participants)
7	**12 months**	**Telephone contact**<br>**Vaccine administration**<br>Synflorix	**Telephone contact**<br>**Vaccine administration**<br>Synflorix

Notes * Each blood sample approximately 5-8mls

The antigens listed shall be given as: DTPa-HepB-Hib-IPV-PRP-T (GlaxoSmithKline),
Each vaccine antigen dose number is signified by subscript numbers. E.g. $D_2T_2Pa_3$ signifies the second vaccination for diphtheria and tetanus toxoids and the third vaccination for the acellular pertussis antigens.

8.2 Medication(s)/treatment(s) permitted (including rescue medication) and not permitted before and/or during the trial.

Any medication is permitted based on clinical indication including those specified in Section 6.3.1.

8.3 Clinical and Laboratory Procedures

8.3.1 Clinical Procedures

The following clinical procedures will be conducted during this study:

- A review of the participant's medical history, including demographics, and concomitant medication
- A brief medical evaluation and other physical examination if clinically indicated.
- Measurement of the participant's axillary temperature.
- A review of the participant's eligibility according to the Inclusion / Exclusion criteria.
- A 5mL blood sample will be collected at visit 1 from the mother and infant blood will be collected at visit 2, 3, 5 and 6 for the determination of antibody titres. The timing and

frequency of the above procedures are described in the Schedule of Assessments (Table 2: Pages 22).

- Participants will be observed for 30 minutes post dPa vaccine at visit 1 and for 15 minutes following vaccinations at visits 2, 4 and 5

8.3.2 Blood Samples for Immunogenicity Analysis

Five blood samples will be collected throughout the study. The day of collection and the volume of blood to be collected is summarised below (Table 3).

Table 3: Blood Collection intervals and volume

Visit Number (Day)		Volume (ml)
Visit 1 (up to day 5)	Maternal blood	5
Visit 2 (Day 42-56)	Infant blood	5
Visit 3 (28-35 days from Visit 2)	Infant blood	5
Visit 5 (Day 176-211)	Infant blood	5
Visit 6 (60-74 days from Visit 5)	Infant blood	5

9 Procedures for monitoring subject compliance.

To ensure that participants receive the correct doses of the vaccines, the study staff must confirm and record the dose volume, batch number and date of administration in the relevant source documentation and then administer the vaccine.

10 Adverse Events

The Investigator at each centre is responsible for ensuring that all AEs and other clinically significant findings are documented and accurately reported. All study staff must understand the requirements and responsibilities related to safety reporting that are outlined below.

10.1 Definitions

10.1.1 Adverse Event (AE)

As per the International Conference on Harmonisation (ICH), an AE is any untoward medical occurrence in a patient or clinical investigation subject administered a pharmaceutical product and which does not necessarily have a causal relationship with this treatment. An AE can, therefore, be any unfavourable and unintended sign (e.g., an abnormal laboratory finding), symptom, or disease temporally associated with the use of a medicinal (investigational) product, whether or not considered related to the medicinal (investigational) product. All AEs must be recorded in the appropriate section of the source document.

<u>Adverse events include:</u>

- Exacerbation (i.e., an increase in the frequency or severity) of a pre-existing condition. Illness present before study entry should be recorded in the medical history section of the source document and only be reported as an AE if there is an increase in the frequency or severity of the condition during the study.
- Intercurrent illnesses with an onset after administration of any one of the study vaccine

a. Solicited Adverse Event

Solicited AEs are those events specifically sought for and recorded by patients in the 7-day diary card.

A solicited AE Diary that will be issued after each vaccination. The diary will constitute source data for this study.

Solicited local AEs that will be collected during this study include: injection site pain, erythema and induration / swelling.

Solicited systemic AEs that will be collected during this study include:
Fever ≥ 37.5°C, reaction such as, drowsiness, periodically more irritable than usual but with normal activity, prolonged crying and refusal to play, prolonged crying and inability to be comforted, anorexia (poor appetite), vomiting judged to be greater than a posset, redness and swelling at the vaccination site each measured in mm, pain scored as none, minor light reaction to touch, crying or protesting to touch, or crying when the leg is moved.

b. Unsolicited Adverse Event

Unsolicited AEs include AEs other than those specifically solicited. Unsolicited AEs will be recorded by the parents/guardians in the Unsolicited AE Diary Card which is issued after each vaccination. The diary will constitute source data for this study. All other medically attended visits between study visits up to visit 6 (8 months old) will be collected

10.1.2 Serious Adverse Event (SAE)

A serious adverse event (SAE) is defined as any adverse event that:

- **Is fatal:** The event must be the cause of death for the SAE to meet the serious criteria of "fatal". (*Note: death is an outcome, not an event*)
- **Is life-threatening:** The term "life-threatening" refers to an event in which the participant was at risk of death at the time of the event; it does not refer to an event, which hypothetically might have caused death if it were more severe.
- **Requires in-patient hospitalisation or prolongation of existing hospitalisation.**
- **Results in persistent or significant disability / incapacity**
- **Is a congenital anomaly / birth defect**
- **Is medically significant according to the investigator's judgement:** Medical and scientific judgement should be exercised when deciding whether an event is serious. Important medical events that may not be immediately life-threatening or result in death or hospitalisation, but may jeopardise the participant or may require intervention to prevent one of the other outcomes listed in the definition above, may be defined as serious.

Note, at least one of the above criteria must be ticked on the SAE page of the source document.

Please note the following may also constitute reporting of an SAE:

- Any SAE (including medically significant events and AESIs), whether or not related to one of the study vaccines, and occurring from Day 0 up to 12 months after recruitment
- Clinical events related to the study conduct that occur after consent but before administration of one of the study vaccines, and which meet the criteria of an SAE

- SAEs considered by the Investigator to be related to one of the study vaccines that occur beyond the defined study period (i.e., more than 12 months after recruitment)

10.2 Assessment of Causality

For this study, all local solicited AEs (injection site pain, erythema and induration / swelling) will be considered definitely related to vaccination.

For all solicited systemic AEs, all unsolicited AEs and all SAEs, the Investigator will need to determine whether the particular event is related to study vaccination. All AEs must be recorded in the CRF.

The relationship of the AE to one of the study vaccinations will be specified as follows:

Not related
A clinical event or laboratory test abnormality

- With a time to study vaccine administration that makes a relationship impossible
- That could be explained by concurrent disease or other drugs / chemical (either patho-physiologically or clinically)
- With a time to vaccine administration that makes a relationship improbable (but not impossible)
- That could also be explained by another equally likely cause
- Where concurrent disease or other drugs / chemicals could provide a plausible explanation

Related
A clinical event or laboratory test abnormality

- That occurs in a plausible time relationship to the administration of the vaccines;
- Where the event is definitive pharmacologically or phenomenologically
- That occurs in a reasonable time relationship to the administration of the vaccine
- Where the event is more likely to be explained by the vaccine than any other cause

10.3 Assessment of Intensity

Intensity of solicited AEs will be graded on a four-point scale (none, mild, moderate, severe), as explained in tables 2 and 3 and reported by parents on the diary cards.

Solicited local AEs are defined and should be graded as follows:

Table 2 Solicited Local AE Definitions and Intensity Grading

Injection Site Reaction	Intensity Grading			
	None (0)	Mild (Grade 1)	Moderate (Grade 2)	Severe (Grade 3)
Pain	Absent	Minor reaction on touch<br>or<br>Does not interfere with daily activities	Cries / protests on touch<br>or<br>Interferes with daily activities	Cries when limb is moved / spontaneously painful<br>or<br>Prevents daily activities
Redness (erythema)	0	≤ 10 mm	> 10 mm to < 30 mm	≥ 30 mm
Induration / swelling	0	≤ 10 mm	> 10 mm to < 30 mm	≥ 30 mm

Solicited systemic AEs are defined and should be graded as follows:

Table 3 Solicited Systemic AE Definitions and Intensity Grading

**Symptom**	**Intensity Grading**			
	**None (0)**	**Mild (Grade 1)**	**Moderate (Grade 2)**	**Severe (Grade 3)**
For all participants				
Fever (axillary temperature)	< 37.5ºC	≥ 37.5ºC to ≤ 38.5ºC	> 38.5ºC to ≤ 39.5ºC	> 39.5 ºC
Vomiting	None	Adverse event easily tolerated by the participant, causing minimal discomfort and does not interfere with everyday activities	Adverse event sufficiently discomforting to interfere with normal everyday activities	Adverse event prevents normal everyday activities or requires significant medical intervention
Diarrhoea	None	Adverse event easily tolerated by the participant, causing minimal discomfort and does not interfere with everyday activities	Adverse event sufficiently discomforting to interfere with everyday activities	Adverse event prevents normal everyday activities or requires significant medical intervention
Feeding	None	Adverse event easily tolerated by the participant, causing minimal discomfort and does not interfere with everyday activities	Adverse event sufficiently discomforting to interfere with everyday activities	Adverse event prevents normal everyday activities or requires significant medical intervention
Irritability	None	Adverse event easily tolerated by the participant, causing minimal discomfort and does not interfere with everyday activities	Adverse event sufficiently discomforting to interfere with everyday activities	Adverse event prevents normal everyday activities or requires significant medical intervention
Drowsiness	None	Adverse event easily tolerated by the participant, causing minimal discomfort and does not interfere with everyday activities	Adverse event sufficiently discomforting to interfere with everyday activities	Adverse event prevents normal everyday activities or requires significant medical intervention
Restlessness	None	Adverse event easily tolerated by the participant, causing minimal discomfort and does not interfere with everyday activities	Adverse event sufficiently discomforting to interfere with everyday activities	Adverse event prevents normal everyday activities or requires significant medical intervention

The intensity / severity of unsolicited AEs will be graded as follows:

Mild: Symptoms are easily tolerated and do not interfere with daily activities
Moderate: Enough discomfort to cause some interference with daily activities
Severe: Symptoms that prevent normal, everyday activities

10.4 Adverse Event Recording

10.4.1 Solicited and Unsolicited Adverse Events

Solicited and unsolicited adverse events (AEs) will be recorded on the diary card by the parents three and six hours after injection and at bedtime each evening for 6 evenings and entered in the eCRF.

Telephone contact will be made with the parents to encourage completion of the diary cards between days 2 and 7 following vaccination. Parents will be issued with cards containing contact details and asked to contact study personnel regarding any perceived serious adverse events. Parents will be telephoned when the subject is 12 months old to collect information on the occurrence of adverse events of special interest, SAE's, new onset of chronic illnesses and associated concomitant medication use and the diagnosis of pertussis infection or other vaccine preventable disease (VPD). Reports of pertussis or other VPD infection between study visits will be confirmed by medical record review of general practitioner or hospital records. The DSMB will review all serious adverse events.

10.4.2 New Onset of Chronic Illness

The new onset of chronic illness will be collected in the same manner as solicited (by diary card used following each vaccination) and unsolicited AEs (reviewed at each study visit) and recorded in the Adverse Event page of the eCRF. These events will be collected until 12 months after recruitment.

10.4.3 Serious Adverse Event (SAE)

SAEs will be recorded in the relevant source document and reported in the AE and SAE layouts of the eCRF. The original SAE reporting form should remain at the study centre with the other source documentation until the close of the study.

10.5 Serious Adverse Event Reporting

All SAEs MUST BE REPORTED to the Principal Investigator (PI) within 24 hours of a site becoming aware of it. Initial notification of an SAE should be made by email or mobile telephone text to the PI immediately. The SAE report with as much information regarding the event that is available at the time should be entered onto the eCRF and the PI notified (fax or email) by close of the next business day.

Each local Investigator will be responsible for reporting individual SAEs and Periodic Safety Updates (as applicable) to the relevant Ethics Committee within the specified time-line.

The Principal Investigator (or other Investigator if so delegated) will report all SAEs (regardless of the centre of origin) causally-related to the monovalent acellular pertussis vaccine to the Chairperson of the DSMB within 24 hours of being informed of the event. The DSMB will convene a DSMB meeting within 24 hours of the SAE notification, either in person or via teleconference, and then make their recommendations to the Principal Investigator.

The Investigator at each study site will report all SAEs (regardless of causality) occurring in infants who have received the monovalent acellular pertussis vaccine to GSK Biologicals, Australia within 24hrs of being informed of the event.
The Investigator at each study site will report all SAEs causally-related to the licensed vaccines to GSK Biologicals, Australia within 24hrs or being informed of the event.

Prompt notification is essential so that regulatory requirements for SAE reporting and ethical obligations to the participants involved in the study can be met. If a telephone report is initially made, it should be followed by a faxed or scanned email copy of the SAE report,

When submitting SAEs, the Investigator should not include the name or the address of the individual participant.

Prospective data collection of adverse events (AEs), including medically attended AEs and significant AEs and serious adverse events (SAEs) will be conducted in accordance with ICH Good Clinical Practice Guidelines.

10.6 Adverse Event Follow-up

Every effort should be made to follow-up participants who continue to experience an AE or an SAE on completion of the study until the AE resolves or stabilises. All follow-up information (and attempted follow-up contacts) should be documented in the participant's records.

10.6.1 The type and duration of the follow-up of subjects after adverse events.

If significant adverse events or contraindications to vaccination are identified, the infant will be managed at the Immunisation Adverse Event Clinics at The Children's Hospital Westmead, Women's and Children's Hospital, Adelaide, or Princess Margaret Hospital for Children or by their usual medical care provider. Significant adverse events shall include those as defined in the Immunisation Handbook or any condition requiring admission to hospital or unexpected medical attendance. Every effort will be made to follow-up subjects who continue to experience an AE or an SAE on study completion until the AE resolves or stabilizes.

10.7 Regulatory Requirements

The sponsors have a legal responsibility to notify the relevant regulatory authorities about the safety of the product under clinical investigation. Prompt notification of SAEs by Investigators is essential so that legal obligations and ethical responsibilities for the safety of other study participants are met.

The Principal Investigator will report all unexpected Serious Adverse Events, causally-related to the monovalent acellular pertussis vaccine, to the Therapeutic Goods Administration (TGA) within 7 days (fatal or life-threatening events) or 15 days (for non-fatal, non-life threatening events).

The Principal Investigator will also report all unexpected or serious adverse reactions causally related to the licensed, concomitant childhood vaccines, to the TGA Office of Medicines Safety Monitoring as soon as possible.

10.8 IEC Reporting Requirements

The time frame within which an IEC must be notified of deaths and study product-related unexpected SAEs is stipulated by each IEC. It is the Investigator's responsibility to comply with the requirements for IEC notification.

The parents/guardians of participants will be provided with the contact details of the study team, and the local HREC for the reporting and management of all adverse events (vaccine and non-vaccine related) that may occur during the study.

10.9 Composition of DSMB

Professor David Isaacs, The Children's Hospital at Westmead
Dr Kristine Macartney, National Centre for Immunisation Research and Surveillance
Dr Jim Buttery, Royal Children's Hospital, Melbourne

The Children's Hospital at Westmead statistician will be a formal member of the DSMB.

11 Assessment of Efficacy

11.1 Specification of the efficacy parameters.

Blood samples will be taken as follows: maternal blood at birth, infant blood at 6 weeks, 10 weeks, 6 and 8 months. We believe that each of these blood samplings is important in the evaluation of antibody kinetics and comparison of the two groups in terms of presumed disease protection.

11.1.1 Antibodies to pertussis:
IgG to PT, pertactin and FHA will be measured by ELISA in maternal and infant serum samples at GSK Biologicals, Belgium, using a validated assay.

11.1.2 Antibodies to other vaccine antigens:
Antibodies to hepatitis B virus, Hib, tetanus, diphtheria, pneumococcal serotypes (all 10 serotypes if size of blood sample permits, if not then priority serotypes are 19A, 6A, 1, 5, 7F, 6B, 23F + Protein D) will be measured on sera taken at 8 months old..

11.1.3 Cellular immune responses –
Cellular immune responses will be measured on samples collected at 10 weeks and 8 months old in participants from Perth and potentially other sites, depending on recruitment. All participants will aim to have approximately 5mls of blood in total collected at visits, 1,2,3, 5 and 6. The first priority is to collect 2-3 mls of blood for antibody measurement, as detailed in *11.1.2*. If an additional 2-3mls of blood is available, then it will be used for cellular immune response measurement. This blood will be collected into an equal volume of RPMI1640 supplemented with 5% pooled AB serum (for cultures with vaccine antigens), or AIM-V serum free medium supplemented with 2-mercaptoethanol ($4x10^{-5}$ M: for cultures with PHA). The following standard stimulation matrix will be employed with all samples: background control, pertussis mix (PT, FHA, PRN), tetanus toxoid (TT; 0.5Lf/ml), hepatitis B antigen (HB; 2.5ug/ml), phytohemagglutinin (PHA; 1ug/ml). TRF will be employed to quantitated cytokine proteins - IFNγ (TH1 immunity) and IL-5 and IL-13 (TH2 immunity). Analysis will be performed at the Institute for Child Health Research, University of Western Australia.

12 Methods and timing for assessing, recording and analysing of efficacy parameters.

Serological samples will be stored frozen prior to transport in batches to GSK Biologicals, Rixensart for serology measurement. The timing of laboratory analysis is to be negotiated with GSK Biologicals and depends on recruitment rate.

Results will be analysed in GSK laboratories in batches depending availability of laboratory time, this will be negotiated with GSK Biologicals.

13 Administrative Aspects

13.1 Initiation of the study

Study personnel at each participating centre will have an initiation visit, performed by the investigator at that site to explain their roles and responsibilities with regard to protocol adherence.

The investigational staff may not enrol any participants prior to receipt of written approval from the Ethics committee, and completion of a formal meeting conducted by sponsors /delegate to initiate the study. This meeting will include an inventory of study supplies and a detailed review of the Protocol and source documentation including participant diary cards.

13.2 Monitoring of the study

The principal investigator will arrange for a suitable qualified Monitor to monitor the study, at regular intervals, at each of the participating centres.

13.3 Protocol Deviation / Violations

All instances where the requirements of the Study Protocol were not complied with will be captured in the relevant source documentation and the Study Monitor will prepare a Protocol Deviation / Violation Log. Corresponding participants may be withdrawn from the study at the discretion of the Investigator. An amendment must be agreed upon by the investigators, but not implemented until written Ethics Committee approval is obtained, except where necessary to eliminate an immediate hazard to study participants or when the change(s) involves only logistical or administrative aspects.
Study Protocol deviations/violations can be defined as follows:

- *Protocol Deviations* arise when participants who have been entered in the Study deviate from the Ethics Committee-approved Study Protocol. For example, a blood sample is not drawn at an interval specified by the Study Protocol.
- *Protocol Violations* arise when participants who did not meet the Inclusion Criteria, or who met Exclusion Criteria are entered into the study.

If a study protocol violation occurs, the Investigator must notify the appropriate Ethics Committee as soon as possible or as per local requirements. Protocol deviations will be noted in the source documentation. All study protocol deviations / violations must be noted in a Protocol Deviation / Violation Log to be maintained in the Investigator's study file at each participating centre.

13.4 Confidentiality

The investigators will preserve the confidentiality of participants taking part in the study. In the event that names inadvertently appear on study documentation, this information will not be processed. Participant medical records pertaining to the study may be inspected / audited at any time by authorised representatives of the sponsors, a regulatory authority or by the Ethics Committee. All records accessed will be strictly confidential. Consent to participate in the study includes consent to these inspections / audits.

14 Statistics

All efficacy analyses will be undertaken using the Full Analysis Set comprising all randomized participants. The safety analyses will be undertaken using the Safety Analysis Set comprising all randomized participants that receive at least one vaccination.

14.1 A description of the statistical methods to be employed, including timing of any planned interim analysis(es).

All serum antibody concentrations will be log transformed for statistical analysis as geometric mean concentrations (GMC). Where protective thresholds are well established, the proportions at or above the assay cut off – anti-diphtheria (0.1 IU/ml)*, anti-tetanus (0.1 IU/ml) anti-Hib (PRP) (0.15 µg/ml) and hepatitis B surface antibody (10mIU/ml) – will be compared by vaccine group. Antibody to polio serotypes 1, 2 and 3 will be measured by microneutralisation assay and the lowest dilution tested at 1:8. Statistical analysis will include both comparisons of GMC (with 95% confidence intervals) as a continuous variable (t test) and categorical analysis of relevant Ab. Thresholds (chi square). Analysis will be performed with the assistance of the NHMRC Clinical Trials Centre, independent of the testing laboratory. *Note: * this threshold is significantly above the known protective level of 0.01 IU/ml but is the lower limit of assay detection*.

The primary analysis will be a comparison in the susceptibility rate (no measurable PRN nor PT) between the two groups at week 10 (or last post-baseline observation carried forward) using a chi-square test. The applicability of more sophisticated analysis approaches that adjust for covariates (e.g. logistic regression) and model the repeated assessments of susceptibility over time Week 6, Week 10, Month 6, Month 8 will be investigated.

The other antibody threshold data collected at Month 8 will be analysed using same approaches as those outlined above for the Week 10 susceptibility data.

The log-transformed antibody concentrations will be analysed using a linear model with treatment group, site and maternal dTpa vaccine status as factors. The applicability of repeated measures analysis methods (e.g. mixed-model) will also be explored for the concentration data.

The analysis of safety data will be primarily descriptive in nature.

14.2 The number of subjects planned to be enrolled.

14.2.1 Antibody measures:

Lack of any measurable antibody to both PT and PRN is the best available indicator of susceptibility to severe pertussis. Accordingly, we have based sample size calculations on reduction in the proportion of susceptible infants as early as possible. If superiority of birth pertussis vaccine is defined as a reduction of 12% or more in the proportion of subjects

lacking antibody (no measurable PRN or PT) compared with controls at 10 weeks old, a sample size of 220 in each maternal arm will have 80% power to detect a reduction of this magnitude at a two-sided alpha level of 0.05. This is not altered by a differing prevalence of detectable pre-immunisation antibody among controls over the range of 65% to 75%. (Sample Power V2 www.spss.com) This study will recruit 110 participants in each of the 4 arms, a total of 440 participants allowing for a 10% drop out rate.

14.2.2 Cell mediated immunity:
Based on our pilot data, a sample size of 22 participants in each of the 4 arms will allow us to detect a difference of greater than 0.25 in log IL-5 with a power of 90% at the alpha level of 0.01. An actual sample size of 25 per group will allow for subject dropouts.

14.3 The level of significance to be used.

Hypothesis tests and confidence intervals will be two-sided with alpha set to 5%.

14.4 Statistical criteria for the termination of the trial.

There are no formal statistical stopping rules for this study.

14.5 Procedure for accounting for missing, unused and spurious data.

From an analysis perspective, a 'drop-out' will be any subject who did not attend the concluding visit. A participant, who returns for the concluding visit in the protocol, even if not all prior visits were attended, is deemed to have completed the study.

Methods for imputing missing values (e.g. last post-baseline observation carried forward) or analysis models that accommodate missing data (e.g. repeated measures mixed-models) will be employed where necessary to ensure as many subjects as possible from the Full Analysis Set contribute to the efficacy analyses.

14.6 The selection of subjects to be included in the analyses

The Full Analysis Set will comprise all randomized participants. The Safety Analysis Set will comprise all randomized participants that receive at least one vaccination

15 Direct Access to Source Data/Documents

The sponsor should ensure that it is specified in the protocol or other written agreement that the investigator(s)/institution(s) will permit trial-related monitoring, audits, IRB/IEC review, and regulatory inspection(s), providing direct access to source data/documents.

The investigators undertake to maintain complete written documentation of trial procedures, which are open to inspection for monitoring and audit by nominated representatives of the sponsor. These records are further open to review by independent ethics committees and regulatory authorities such as the Therapeutic Goods Administration.

16 Quality Control and Quality Assurance

Recruitment in an acceptable time frame requires multi-centre collaboration for a study of this type. All investigators have extensive experience in the conduct of infant vaccine trials, including recruitment at birth and are confident of targets of 110 subjects per centre. The trial

groups in Sydney, Melbourne, Adelaide and Perth have extensively collaborated in research level over the last ten years.

17 Ethics

This study will be conducted according to Good Clinical Practice as defined by the International Conference on Harmonisation. The study protocol will vary from standard immunisation practices only in the earlier provision of Pa vaccine to newborn infants in group 1 and the administration of the first dose of Pa containing vaccine at 6 weeks old in infants in both groups, replacement of Prevenar with Synflorix and the collection of blood samples. The infants will each receive immunisation for all age-appropriate antigens specified in the Australian Immunisation Handbook (currently 9th edition [15]) except that the first standard vaccine will be given from 6 weeks of age, two weeks earlier than the current schedule age of 8 weeks (2 months). The Pa combination vaccine, used at this age, is licensed for use from 6 weeks old and is considered a valid dose by ACIR. The infants will be under the care of medical and nursing staff expert in the administration of vaccinations to infants and the collection of blood samples from infants. There are few data on responses to DTPa vaccines in infants younger than 6 weeks. There are no grounds to suggest earlier administration will be harmful (see background for further discussion).

18 Data Handling and Record Keeping

Each study centre will maintain an individual file and source documentation for every participant enrolled in the study. The file will include visit dates of the participant, along with relevant demographic information. The file will further document participant eligibility for the study as well as any serious adverse reactions experienced and other notes as appropriate. An electronic CRF must be completed for each enrolled participant and will be checked against the source documents to ensure data completeness and accuracy as required by the study protocol.

The eCRF and source documents will be kept in order and up to date so that they always reflect the latest observations on the participants enrolled in the study. The source documents will be securely stored in locked cabinets and will only be accessed by appropriately trained staff at each study centre and the electronic eCRF will comply with applicable privacy laws.

The original, signed consent form will be filed with each participant's source documentation.

All eCRFs must be signed off by the local Investigator (or their delegate) on completion of the study.

All study forms for each enrolled infant will be stored securely. Data will be entered into a database, designed to have appropriate data checks. Access to databases will be restricted by password. Back up copies of the forms and database will be made and stored separately. Data will not be collected from any federal government agency e.g. Medicare. Data will be stored securely for a period up to 30 years or as required by standard trial procedures.

19 Financing and Insurance

GSK Biologicals has agreed to supply the monovalent acellular pertussis and Synflorix vaccines and perform antibody measurement and has a clinical trial agreement with each of the four study centres.

This trial is supported by an NHMRC project grant (No 570756)

Each site will indemnify its own staff engaged in study conduct according to existing local arrangements.

20 References

1. National Centre for Immunisation Research and Surveillance of Vaccine Preventable Diseases. Vaccine preventable diseases and vaccination coverage in Australia, 2003-2005. *CommunDisIntell* 2007; 31: S1-152.

2. Tan T, Trindale E, Skowronski D. Epidemiology of pertussis. *Pediatr Infect Dis J.*2005; 24: S10-18.

3. Tanaka M, Vitek C, Pascual F et al. Trends in pertussis among infants in the United States, 1980-1999. *JAMA* 2003; 23: 2968-2975.

4. Elliot E, McIntyre P, Ridley G et al. National study of infants hospitalised with pertussis in the acellular pertussis vaccine era. *Pediatr Infect Dis J* 2004; 23: 246-252.

5. Juretzko P, Fabian-Marx T, Haastert S, Giani G, von Kries R, Wirsing von Konig CH. Pertussis in Germany: regional differences in management and vaccination status of hospitalized cases. *Epidemiology and Infection* 2001; 127:63-71.

6. Crowcroft NS, Andrews N, Rooney C, Brisson M, Miller E. Deaths from pertussis are underestimated in England. *Arch Dis Child* 2002; 86:336-8.

7. Crowcroft NS, Pebody R. Recent developments in pertussis. *Lancet* 2006; 367: 1926-1936.

8. Wood N McIntyre P. Pertussis in infants: preventing deaths and hospitalisations in the very young. *J PaedsChild Hlth* 2008 in press; accepted March 5th 2008.

9. Hull B, McIntyre P. Timeliness of childhood immunisation in Australia. *Vaccine* 2006; 24: 4403-4408.

10. World Health Organization Immunization policy: Global Programme for Vaccines and Immunization - Expanded Programme on Immunization. WHOGPV/GEN/95.03 Rev.1. Geneva: World Health Organization; 1996.

11. Forsyth K, Tan T, Wirsing Von Kirsig C et al. Potential strategies to reduce the burden of pertussis. *Pediatr Infect Dis J* 2005; 24: S69-74.

12. McIntyre P. Vaccination strategies for the prevention of neonatal pertussis. *Expert Rev Vaccines* 2004; 34: 375-378.

13. Mooi F, de Greeff S. The case for maternal vaccination against pertussis. *Lancet Infect Dis* 2007; 7: 614-62.

14. Van Rie A, Wendelboe A, Englund J. Role of maternal pertussis antibodies in infants. *Pediatr Infect Dis* 2005; 24: S62-65.

15. National Health and Medical Research Council: *The Australian immunisation handbook*. 9thedn. Australian Government Department of Health and Ageing, Canberra, 2008.

16. Halsey N, Galazka A. The efficacy of DPT and oral poliomyelitis immunization schedules initiated from birth to 12 weeks of age. *Bulletin of WHO* 1985; 63:1151-69.

17. Provenzano RW, et al Immunization and antibody response in the newborn infant. I. Pertussis inoculation within twenty-four hours of birth. *New Engl J Med* 1965; 273: 959-961.

18. Booy R, Aitken SJ, Taylor S, et al. Immunogenicity of combined diphtheria, tetanus, and pertussis vaccine given at 2, 3, and 4 months versus 3, 5, and 9 months of age. *Lancet* 1992; 339 : 507-10.

19. Baraff LJ, et al. Immunologic response to early and routine DTP immunization in infants. *Pediatrics* 1984; 73:37-42.

20. Belloni C, De Silvestri A, Tinelli C, Avanzini MA, Marconi M et al. Immunogenicity of a three-component acellular pertussis vaccine administered at birth. *Pediatrics* 2003; 111:1042-5.

21. Knuf M, Schmitt H, Wolter J et al. Neonatal vaccination with an acellular pertussis vaccine accelerates the acquisition of pertussis antibodies in infants. *J Pediatrics* 2008 in press.

22. Halasa N, O'Shea A, Lafleur B. Safety and Immunogenicity of an additional dose of DTPa given at birth. Abstract: 45th Annual Interscience Conference on Antimicrobial Agents and Chemotherapy, 2005, Washington DC, USA.

23. McIntyre P, Wood N, Marshall H, Roberton D. Acellular pertussis vaccine at birth: evidence of immunogenicity. Abstract: *47th Annual Interscience Conference on Antimicrobial Agents and Chemotherapy*, 2007, Chicago, USA.

24. McVernon J, Andrews N, Slack MP et al. Risk of vaccine failure after Haemophilus influenzae type b (Hib) combination vaccines with acellular pertussis. *Lancet*. 2003; 361:1521-3.

25. Olin P, Gustafsson L, Barreto L et al. Declining pertussis incidence in Sweden following the introduction of acellular pertussis vaccine. *Vaccine* 2003; 21: 2015-2021.

26. Siegrist C-A. Neonatal and early life vaccinology. *Vaccine* 2001; 19: 3331-46.

27. Miller E, Ashworth LA, Redhead K, Thornton C, Waight PA, Coleman T. Effect of schedule on reactogenicity and antibody persistence of acellular and whole-cell pertussis vaccines: value of laboratory tests as predictors of clinical performance. *Vaccine* 1997;15:51-60.

28. Salmaso S, Mastrantonio P, Tozzi AE, et al. Sustained efficacy during the first 6 years of life of 3-component acellular pertussis vaccines administered in infancy: the Italian experience. Pediatrics 2001;108:e81
