# Supplementary material for: Immunogenicity and Safety of Monovalent Acellular Pertussis Vaccine at Birth: A Randomized Clinical Trial
Source: JAMA Pediatr. 2018 Sep 10;172(11):1045–52. doi: 10.1001/jamapediatrics.2018.2349 (PMC6248137; doi:10.1001/jamapediatrics.2018.2349)
Supplement: Supplement 2. — eAppendix. Supplementary Methods eTable 1. Pertussis Toxin IgG Antibody Levels by Maternal Prepregnancy Tdap Vaccine History and Receipt of aP Vaccine at Birth eTable 2. Filamentous Haemaglutinin IgG Antibody Levels by Maternal Prepregnancy Tdap Vaccine History and Receipt of aP Vaccine at Birth eTable 3. Pertactin IgG Antibody Levels by Maternal Prepregnancy Tdap Vaccine History and Receipt of aP Vaccine at Birth eTable 4. Systemic and Local Reactions Within 2 Days After Birth Dose of Acellular Pertussis Vaccine Compared With Controls [file jamapediatr-172-1045-s002.pdf]

Supplementary Online Content

Wood N, Nolan T, Marshall H, et al. Immunogenicity and safety of monovalent acellular pertussis vaccine at birth: a randomized clinical trial. *JAMA Pediatr.* Published online September 10, 2018. doi:10.1001/jamapediatrics.2018.2349

eAppendix. Supplementary Methods

eTable 1. Pertussis Toxin IgG Antibody Levels by Maternal Prepregnancy Tdap Vaccine History and Receipt of aP Vaccine at Birth

eTable 2. Filamentous Haemaglutinin IgG Antibody Levels by Maternal Prepregnancy Tdap Vaccine History and Receipt of aP Vaccine at Birth

eTable 3. Pertactin IgG Antibody Levels by Maternal Prepregnancy Tdap Vaccine History and Receipt of aP Vaccine at Birth

eTable 4. Systemic and Local Reactions Within 2 Days After Birth Dose of Acellular Pertussis Vaccine Compared With Controls

This supplementary material has been provided by the authors to give readers additional information about their work.

© 2018 Wood N et al. *JAMA Pediatrics.*

eAppendix. Supplementary Methods

Description of vaccines and vaccine procedures: A single dose of investigational aP (0.5mL) vaccine containing pertussis toxin (PT) 25µg, pertactin (PRN) 8 µg, filamentous haemagglutinin (FHA) 25 µg and 0.5 mg aluminium as hydroxide salts was supplied by GlaxoSmithKline (GSK) Vaccines, Rixensart, Belgium. All infants received 10µg hepatitis B surface antigen (HbsAg) with 0.25mg aluminium hydroxide adjuvant (*Engerix B®*). The aP vaccine was administered intramuscularly into the right anterolateral thigh prior to 120 hours of age. Due to local procedures, some participants were given HBV vaccine prior to receipt of aP vaccine, in others aP (right thigh) and HBV (left thigh) vaccines were given concomitantly.

Infants received the following vaccines :
Infanrix hexa – GlaxoSmithKline Australia Pty Ltd (DTPa-hepB-IPV-Hib; diphtheria-tetanus-acellular pertussis-hepatitis B-inactivated poliovirus-Haemophilus influenzae type b). The vaccine consists of both a 0.5 mL pre-filled syringe containing ≥30 IU diphtheria toxoid, ≥40 IU tetanus toxoid, 25 µg PT, 25µg FHA, 8 µg PRN, 10 µg recombinant HBsAg, 40 D-antigen units inactivated poliovirus type 1 (Mahoney), 8 D-antigen units type 2 (MEF-1) and 32 D-antigen units type 3 (Saukett), adsorbed onto aluminium hydroxide/phosphate; traces of formaldehyde, polysorbate 80, polysorbate 20, polymyxin and neomycin; and a vial containing a lyophilised pellet of 10 µg purified Hib capsular polysaccharide (PRP) conjugated to 20–40 µg tetanus toxoid

Synflorix – GlaxoSmithKline Australia Pty Ltd (10-valent pneumococcal conjugate vaccine; 10vPCV). Each 0.5 mL monodose vial or pre-filled syringe contains 1 µg of pneumococcal capsular polysaccharide of serotypes 1, 5, 6B, 7F, 9V, 14, 23F and 3 µg of serotype 4, conjugated to a total of 9–16 µg of non-typeable H. influenzae protein D, 3 µg of serotype 18C conjugated to 5–10 µg of tetanus toxoid carrier protein, and 3 µg of serotype 19F conjugated to 3–6 µg of diphtheria toxoid carrier protein, adsorbed onto 0.5 mg aluminium as aluminium phosphate

Rotarix – GlaxoSmithKline (live attenuated RIX4414 human rotavirus strain, type G1P1A[8]). Each 1.5 mL monodose pre-filled oral applicator or squeezable tube contains ≥106.0 cell culture infectious dose 50% (CCID50) of the RIX4414 strain; di-sodium adipate; Dulbecco's Modified Eagle Medium; sterile water. Manufacture involves exposure to bovine-derived material. (Sydney particpants only)

RotaTeq – CSL Limited/Merck & Co Inc (live attenuated human–bovine reassortant rotavirus strains, types G1, G2, G3, G4 and P1A[8]). Each 2.0 mL monodose pre-filled dosing tube contains a minimum dose level of at least 2.0 × 106 infectious units of each of the rotavirus reassortants G1, G2, G3, G4 and P1A[8]; sodium citrate; sodium phosphate monobasic monohydrate; sodium hydroxide; polysorbate 80; cell culture medium. Manufacture involves exposure to bovine-derived material. (Melbourne, Adelaide, Perth participants)

Laboratory analysis and serology measurement
Samples were centrifuged, serum separated, stored at -80ºC and shipped frozen to GSK Biologicals, Belgium (GSK) where blinded serologic assays (enzyme linked immunosorbent assay (ELISA)) were performed for all vaccine antibody responses (pertussis and other vaccine antigens).

Pertussis toxin (anti-PT), pertactin (anti-PRN), and filamentous hemagglutinin (anti-FHA) IgG antibody concentrations were measured at each sampling point by enzyme linked immunosorbent assay (ELISA: cut-off 5 EL.U/mL) using standard assay methods at the GSK laboratory developed for licensure of DTPa vaccines.

© 2018 Wood N et al. *JAMA Pediatrics.*

Anti-diphtheria (cut-off 0.1 IU/mL), anti-tetanus (cut-off 0.1 IU/mL), and anti-PRP (cut-off 0.15 ug/mL) and anti-pneumococcal IgG antibodies were measured by ELISA on the sample taken at 32 weeks of age (8 weeks after the final vaccine dose). Hepatitis B surface antibodies (anti-HBs) were measured by ELISA (cut-off 10 mIU/mL) on samples collected at 32 weeks of age. There was no formal surveillance for pertussis infection.

Adverse event monitoring and data collection

Parents were given a thermometer, and asked to record temperature and any solicited adverse reactions 3 and 6 hours after injection and at bedtime each evening for 7 days. Solicited systemic adverse reactions included: fever, drowsiness (unusually sleepy or inactive), irritability, anorexia, vomiting, redness and swelling at the vaccination site (each measured in millimetres) and pain. Systemic adverse reactions were graded according to severity.

© 2018 Wood N et al. *JAMA Pediatrics.*

eTable 1. Pertussis Toxin IgG Antibody Levels by Maternal Prepregnancy Tdap Vaccine History and Receipt of aP Vaccine at Birth

	**Maternal Tdap within 5 years of enrolment**					**No Maternal Tdap within 5 years of enrolment**				
	**aP group**		**Control**			**aP group**		**Control**		
**Age**	% above detectable	GMC^ (95% CI)	% detectable	GMC^ (95% CI)	P value	% above detectable	GMC^ (95% CI)	% above detectable	GMC^ (95% CI)	P value
**Baseline (birth)**	89.8	14.49 (10.9,19.2)	80.4	13.57 (9.92,18.58)	0.76	54.5	6.45 (5.55,7.5)	49.1	5.46 (4.76,6.27)	0.11
**6 weeks**	82.2	11.13 (8.4,14.74)	64.4	8.63 (6.24,11.93)	0.25	60	6.69 (5.84,7.66)	29.4	4.04 (3.55,4.6)	<.0001
**10 weeks**	93.6	19.28 (15.36,24.21)	70.5	7.45 (5.69,9.75)	<.0001	98.1	27.59 (24.16,31.51)	54.4	5.68 (4.95,6.51)	<.0001
**6 months**	100	35.53 (29.36,42.99)	100	26.64 (22.08,32.15)	0.04	100	44.86 (40.31,49.93)	99.3	33.83 (30.71,37.27)	0.0002
**8 months**	100	44.2 (35.72,54.7)	100	35.48 (29.46,42.71)	0.14	100	55.62 (50.35,61.44)	100	47.93 (43.85,52.39)	0.03

Abbreviations: TdaP, low dose adult formulated diphtheria, tetanus and acellular pertussis vaccine, ^ GMC – geometric mean concentration (EL.U/mL), aP group – monovalent aP and hepatitis B vaccine at birth, then Infanrix Hexa® at 6 weeks, 4 and 6 months of age, Control – hepatitis B vaccine at birth then Infanrix Hexa® at 6 weeks, 4 and 6 months of age, 95% CI, 95% confidence interval

© 2018 Wood N et al. *JAMA Pediatrics.*

eTable 2. Filamentous Haemaglutinin IgG Antibody Levels by Maternal Prepregnancy Tdap Vaccine History and Receipt of aP Vaccine at Birth

	**Maternal Tdap < 5 years of enrolment**					**No Maternal Tdap < 5 years of enrolment**				
	**aP group**		**Control**			**aP group**		**Control**		
**Age**	% above detectable	GMC^ (95% CI)	% detectable	GMC^ (95% CI)	P value	% above detectable	GMC^ (95% CI)	% above detectable	GMC^ (95% CI)	P value
**Baseline (birth)**	100	127.98 (98.6,166.2)	91.3	100.99 (66.3,153.4)	0.35	92.8	25.58 (21.3, 30.7)	88.5	19.78 (16.6, 23.5)	0.05
**6 weeks**	100	102.79 (78.6,134.4)	91.3	71.47 (47.3,108.03)	0.16	99.4	26.69 (23.6, 30.2)	76.7	12.98 (10.7,15.7)	<.0001
**10 weeks**	100	104.11 (85.7,126.5)	97.7	55.38 (40.5,75.7)	0.0011	100	127.98 (112.4,145.7)	98	22.14 (19.5, 25.1)	<.0001
**6 months**	100	146.17 (121.6,175.7)	100	90.07 (75.2, 107.9)	0.0005	100	207.19 (187,229.6)	100	140.9 (125.1, 158.8)	<.0001
**8 months**	100	204.42 (168.3,248.3)	100	150.66 (123.2,184.3)	0.04	100	279.7 (254.5,307.4)	100	236.8 (215.1, 260.6)	0.02

Abbreviations: TdaP, low dose adult formulated diphtheria, tetanus and acellular pertussis vaccine, ^ GMC – geometric mean concentration (EL.U/mL), aP group – monovalent aP and hepatitis B vaccine at birth, then Infanrix Hexa® at 6 weeks, 4 and 6 months of age, Control – hepatitis B vaccine at birth then Infanrix Hexa® at 6 weeks, 4 and 6 months of age, 95% CI, 95% confidence interval

© 2018 Wood N et al. *JAMA Pediatrics.*

eTable 3. Pertactin IgG Antibody Levels by Maternal Prepregnancy Tdap Vaccine History and Receipt of aP Vaccine at Birth

	Maternal Tdap < 5 years of enrolment					No Maternal Tdap < 5 years of enrolment				
	aP group		Control			aP group		Control		
**Age**	% above detectable	GMC^ (95% CI)	% detectable	GMC^ (95% CI)	P value	% above detectable	GMC^ (95% CI)	% above detectable	GMC^ (95% CI)	P value
**Baseline (birth)**	98	69.98 (47.86,102.31)	91.3	38.36 (24.8,59.33)	0.05	49.1	8.2 (6.58,10.24)	51.7	7.09 (5.86,8.57)	0.33
**6 weeks**	95.6	46.67 (31.37,69.41)	78.3	24.78 (15.73,39.05)	0.04	55.8	7.32 (6.03,8.87)	35.1	5.13 (4.25,6.19)	0.01
**10 weeks**	100	37.61 (27.98,50.54)	84.1	20.76 (14.34,30.06)	0.02	96.2	30.07 (25.31,35.73)	79.9	12.59 (10.57,15)	<.0001
**6 months**	95.5	28.95 (21.73,38.57)	94.9	19.43 (14.11,26.76)	0.07	98.7	51.04 (44.24,58.89)	98.6	44.49 (37.33,53.01)	0.23
**8 months**	95.1	66.1 (46.93,93.11)	97.2	52.06 (38.71,70.01)	0.32	100	96.56 (84.98,109.72)	99.3	88.35 (77.26,101.02)	0.35

Abbreviations: TdaP, low dose adult formulated diphtheria, tetanus and acellular pertussis vaccine, ^ GMC – geometric mean concentration (EL.U/mL), aP group – monovalent aP and hepatitis B vaccine at birth, then Infanrix Hexa® at 6 weeks, 4 and 6 months of age, Control – hepatitis B vaccine at birth then Infanrix Hexa® at 6 weeks, 4 and 6 months of age, 95% CI, 95% confidence interval

© 2018 Wood N et al. *JAMA Pediatrics.*

eTable 4. Systemic and Local Reactions Within 2 Days After Birth Dose of Acellular Pertussis Vaccine Compared With Controls

**Adverse event**	**Severity***	**Participants with local and systemic reactions n (%)**	
**Local reactions**		**aP at birth (n=221 (%))**	**Control (n=219#) (%)**
Pain	Any	41/208 (20%)	14/150 (9%)
	Severe	1/208 (0.5%)	0
Erythema	Any	57/208 (27%)	30/150 (20%)
	Severe	0	0
Swelling	Any	26/208 (12.5%)	6/150 (4%)
	Severe	0	0
**Systemic reactions**			
Irritability	Any	54/221 (24%)	21/103 (20%)
	Severe	2/221(0.9%)	1/103 (0.9%)
Vomiting	Any	45/221 (20%)	24/103 (23%)
	Severe	0	0
Diarrhoea	Any	38/221 (17%)	12/103 (12%)
	Severe	0	0
Feeding	Any	28/221 (13%)	17/103 (17%)
	Severe	0	1/103 (0.9%)
Drowsiness	Any	39/221 (18%)	29/103 (28%)
	Severe	1/221 (0.5%)	1/103 (0.9%)
Restlessness	Any	49/221 (22%)	32/103 (31%)
	Severe	2/221 (1%)	3/103 (3%)
Fever	≥38ºC	0	1/138 (0.7%)
	≥39ºC	0	0

Abbreviations**:** aP at birth group – monovalent aP and hepatitis B vaccine at birth, then Infanrix Hexa® at 6 weeks, 4 and 6 months of age, Control – hepatitis B vaccine at birth then Infanrix Hexa® at 6 weeks, 4 and 6 months of age
Definitions

- #Data on adverse events within 2 days following birth hepatitis B vaccine was only available for a subset of Control participants. This is because some participants had received hepatitis B vaccine more than 2 days before study enrolment and adverse events data was not requested.

***Severity grading of reactions**
Systemic reactions – Any = Grade1, 2, 3, Severe = Grade 3
Grade 1 severity - Adverse event easily tolerated by the participant, causing minimal discomfort and does not interfere with everyday activities
Grade 2 severity - Adverse event sufficiently discomforting to interfere with everyday activities
Grade 3 severity - Adverse event prevents normal everyday activities or requires significant medical intervention
Fever ºC – Fever in degrees celsius

Local reactions
Any = grade1, 2, 3, Severe = grade 3
Erythema and swelling – Grade 1 = <10mm, Grade 2 = .10 to <30mm, Grade 3 = ≥30mm
Pain - Grade 1 = Minor reaction on touch or does not interfere with daily activities, Grade 2= cries / protests on touch or Interferes with daily activities, Grade 3 = Cries when limb is moved / spontaneously painful or prevents daily activities

© 2018 Wood N et al. *JAMA Pediatrics.*
